# Fermatean fuzzy copula aggregation operators and similarity measures-based complex proportional assessment approach for renewable energy source selection

**DOI:** 10.1007/s40747-022-00743-4

**Published:** 2022-05-10

**Authors:** Arunodaya Raj Mishra, Pratibha Rani, Abhijit Saha, Tapan Senapati, Ibrahim M. Hezam, Ronald R. Yager

**Affiliations:** 1 Department of Mathematics, Government College Raigaon, Satna, MP India; 2 Department of Mathematics, Rajiv Gandhi National Institute of Youth Development, Sriperumbudur, TN India; 3 Department of Mathematics, Techno College of Engineering Agartala, Maheshkhola, Tripura 799004 India; 4 Department of Mathematics, Padima Janakalyan Banipith, Kukrakhupi, Jhargram, 721517 India; 5 Department of Statistics and Operations Research, College of Sciences, King Saud University, Riyadh, Saudi Arabia; 6 Machine Intelligence Institute, Iona College, New Rochelle, NY 10801 USA

**Keywords:** Renewable energy, Fermatean fuzzy set, Archimedean copula, Maclaurin mean operator, Similarity measure, COPRAS

## Abstract

Selecting the optimal renewable energy source (RES) is a complex multi-criteria decision-making (MCDM) problem due to the association of diverse conflicting criteria with uncertain information. The utilization of Fermatean fuzzy numbers is successfully treated with the qualitative data and uncertain information that often occur in realistic MCDM problems. In this paper, an extended complex proportional assessment (COPRAS) approach is developed to treat the decision-making problems in a Fermatean fuzzy set (FFS) context. First, to aggregate the Fermatean fuzzy information, a new Fermatean fuzzy Archimedean copula-based Maclaurin symmetric mean operator is introduced with its desirable characteristics. This proposed operator not only considers the interrelationships between multiple numbers of criteria, but also associates more than one marginal distribution, thus avoiding information loss in the process of aggregation. Second, new similarity measures are developed to quantify the degree of similarity between Fermatean fuzzy perspectives more effectively and are further utilized to compute the weights of the criteria. Third, an integrated Fermatean fuzzy-COPRAS approach using the Archimedean copula-based Maclaurin symmetric mean operator and similarity measure has been developed to assess and rank the alternatives under the FFS perspective. Furthermore, a case study of RES selection is presented to validate the feasibility and practicality of the developed model. Comparative and sensitivity analyses are used to check the reliability and strength of the proposed method.

## Introduction

Energy is one of the significant inputs in our lives. It has become an essential pillar for the sustainable growth and well-being of any country at the present time. Due to the continuing urbanization and growth of the world’s population, the worldwide use of energy has risen exponentially. Current conventional sources are not at an adequate level to meet the world’s energy demand for 30–40 years. In parallel with energy consumption, “*greenhouse gas *(*GHG*)” emissions and environmental problems such as air pollution and global warming are also rapidly increasing in our atmosphere. The scarcity of conventional energy sources, as well as their negative environmental consequences, is driving up demand for “*renewable energy sources *(*RESs*)”. Renewable energy causes less GHG effect and replenishes itself over a period of time without depleting the Earth’s resources. To meet sustainable development goals, several countries have paid increased attention to RESs to address the issues related to the ecological problems and energy crisis. Renewable energy sources (RESs) are now a significant component of the economic growth of many countries, with the purpose of avoiding global warming emissions and promoting clean and safe development.

Due to several conflicting evaluation criteria, the selection of the best RES can be treated as a “*multi-criteria decision-making* (*MCDM*)” problem. MCDM is a tool that is employed to choose the desirable option from a set of options by assessing them over a range of criteria. MCDM techniques enable us to assess the candidates and make a selection among them. Decisions on practical RES selection are usually made in uncertain contexts because of numerous factors, such as partial ignorance, incomplete data, or inaccurate decision information. Moreover, the human assessment of qualitative criteria is often vague and subjective. These types of decisions are hard to model with simple numbers. Instead, linguistic variables are used to show how people make decisions that are ambiguous, vague, and subjective.

Certain uncertainties and fuzziness are common attributes of decision making [59]. To deal with an uncertain environment, the decision support system is facing issues regarding the assessment of RESs [45, 66]. Related work on this topic is emerging in an endless stream, especially research on uncertainty-based decision making and other methods, such as the event triggered approach [17], adaptive dynamic programming [25], PD-Type Iterative Learning [96], asynchronous fault detection for 2-D and interval type-2 fuzzy nonhomogeneous higher level Markov jump systems [18, 93], “*fuzzy inference system *(*FIS*)”-based “*analytical hierarchical process *(*AHP*)” making [59], non-cooperative game method [89], advanced integrated multi-dimensional evaluation approach [79], “*intuitionistic fuzzy goal programming *(*IFGP*)” approach [35], Kuhn–Tucker optimization technique for the optimal global solution [14], emended min–max method-based interactive bi-objective optimization algorithm [30] and SWARA–CoCoSo [63] and others.

To cope with the uncertainty of human preferences, Zadeh [91] originated the doctrine of “*fuzzy sets *(*FSs*)”. A fuzzy number is capable of expressing linguistic variables and reducing human error in the decision-making process. Over the past decades, several real-life uncertainties have been effectively handled by FS theory. With the use of Zadeh’s FS theory, many researchers have worked rigorously on the RES selection problem. To avoid the shortcoming of FS, Atanassov [8] suggested the notion of “*intuitionistic fuzzy set* (*IFS*)”, as a generalized version of FS theory. It is represented by the “*membership degree* (*MD*)” and “*non-membership degree* (*NMD*)”, and assures a constraint that the sum of the MD and NMD is $\le 1.$ It has been observed that the IFS theory is one of the most successful and potent tools for managing vague, uncertain, and imprecise information. After the pioneering work of Atanassov [8], several aggregation operators, decision-making techniques, and information measures have been successfully introduced within the context of IFS. Later, Yager [88] extended the IFSs to the “*Pythagorean fuzzy sets* (*PFSs*)” theory. It assures a condition that the square sum of MD and NMD is $\le 1.$ The scope of MD and NMD enlarges in PFS theory [88], i.e., the “*decision making experts *(*DMEs*)” make their decisions more liberally under the PFS setting. For the further improvement of IFS and PFS, Senapati & Yager [73] initiated the theory of “*Fermatean fuzzy set* (*FFS*)”, which satisfies that the cube addition of MD and NMD is restricted to unity. The theory of FFS employs new definitions that make them more efficient and more flexible than IFSs and PFSs in managing uncertain data.

### Research gap and motivation of the study

The flexibility of FFSs may allow us to solve the problems with a higher level of uncertainty, which makes the process more intelligent. Thus, using the Fermatean fuzzy-based framework can lead to a higher accuracy in the evaluation of alternatives in the MCDM procedure. Consequently, the present study focuses on the FFS context. The measure of similarity is an important aspect for disease diagnosis, image segmentation, texture analysis, and a variety of other real-world problems. In view of that, it is very important and essential to study the similarity measure for FFSs. Very few researchers have been engaged in the development of Fermatean fuzzy similarity measures and their significance [10]. Thus, this study proposes new similarity measures for FFSs.

Furthermore, the best RES selection is one of the supreme disciplines in realistic situations. Despite the fact that numerous decision-making methods have been successfully implemented in the complex RES selection process, no research has been conducted on the FFSs-based MCDM model for selecting the best RES candidate(s). Consequently, it is important and interesting to develop an innovative decision-making method to find the solution to the RES selection problem under a Fermatean fuzzy environment.

“*Aggregation operators* (*AOs*)” are mathematical functions, but they are implemented as very simple procedures that combine all of the input individual information into an aggregated one. In the past few years, copious researchers have shown their interest in inquisition on AOs due to their large impact in the area of an ample range of information handling, namely, pattern recognition, decision-making, medical diagnosis, information retrieval, machine learning, data analysis, and others. We may remind you here that the study of Fermatean fuzzy AOs has been drawing noteworthy attention from the authors because of its significance for information fusion. Fermatean fuzzy weighted algebraic and geometric AOs [71, 73], Fermatean fuzzy Dombi weighted algebraic and geometric AOs [9], Fermatean fuzzy Hamacher weighted algebraic and geometric AOs [36], and Fermatean fuzzy Einstein weighted algebraic and geometric AOs [64] have been utilized so far for aggregating data with FFSs. However, none of them can capture the interrelationships among the criteria. In reality, sometimes two or more than two criteria are correlatively dependent. Along these lines, there is as yet an earnest need for an aggregation operator that considers the interrelationships among multi-input criteria under an FFS setting.

The “*complex proportional assessment* (*COPRAS*)” [92] is one of the most significant and well-known MCDM approaches for the ranking of given alternatives by means of numerous conflicting criteria. Recently, the COPRAS methodology has been generalized from different perspectives. However, the classical COPRAS approach fails to handle the RES selection process with an FFS setting. Hence, to improve the flexibility of the COPRAS method, this study develops an extended COPRAS method under an FFS setting.

In the process of decision analysis, the computation of criteria weights is very important as this directly influences the result of the entire assessment and selection procedure. In view of that, this study proposes a novel Fermatean fuzzy similarity measure-based formula to determine the criteria weights.

### Contributions of this study

The current research is an attempt to provide an operational and strategic decision support system for selecting the most appropriate RES among a set of alternative sources. The proposed framework would be efficient by the application of an integrated Fermatean fuzzy COPRAS method by combining the Archimedean copula based Maclaurin symmetric mean operator and similarity measure within the FFS context. The key contributions to this work are as follows:

(i) Novel similarity measures have been developed for FFSs. In addition, a new weighting procedure based on similarity measures is proposed to determine the criteria weights.
(ii) Inspired by Archimedean copula operations and the “Maclaurin symmetric mean (MSM)” operator, this study develops an Archimedean copula-based Maclaurin symmetric mean operator to aggregate the FFSs.
(iii) An integrated COPRAS methodology is introduced with the combination of the proposed copula Maclaurin mean operator and a similarity measure-based weighting procedure on FFSs setting.
(iv) To test the applicability of the presented FF-COPRAS method, a case study of renewable energy source selection in Gujarat, India, is presented.
(v) To prove the reliability and robustness of the present methodology, comparative and sensitivity analyses are executed.

### Structure of this study

The rest of this article is sorted as: “Literature review” offers a comprehensive review of this work. “Proposed similarity measures for FFSs” provides the basis ideas of FFSs. “Proposed Fermatean fuzzy Archimedean copula MSM operator” proposes an Archimedean copula based MSM operator for aggregating the FFSs setting, and furthermore, two novel similarity measures are developed to enumerate the degree of similarity between FFSs. “Proposed Fermatean fuzzy COPRAS (FF-COPRAS) method” introduces an extended COPARS method based on the new Archimedean copula Maclaurin mean operator, score function, and similarity measure under the FFS context. In “Case study: renewable energy source (RES) selection”, the developed method is employed to select the most suitable RES candidate with Fermatean fuzzy information (FFI). Furthermore, comparison with extant approaches and sensitivity investigation are performed. In addition, “Case study: renewable energy source (RES) selection” shows the discussion and recommendations for RESs. Finally, “Conclusions” presents the conclusions and recommendations for further research.

## Literature review

In this part of the study, we present a detailed literature review associated with this study.

### Renewable energy source (RES) selection

The selection of the optimal RES candidate is a more decisive and strategic area. A substantial amount of research has been conducted in the literature to estimate the performance of RESs in various uncertain contexts. These studies utilized a range of decision-making techniques to rank the RESs alternatives. Yuan et al. [90] established an innovative MCDM model by combining Choquet integral and linguistic hesitant fuzzy sets for assessing the RESs in Jilin, China. Mishra et al. [58] suggested an intuitionistic fuzzy information based framework for RESs evaluation. In accordance with the “*decision-making trial and evaluation laboratory* (*DEMATEL*)” and “*technique for order of preference by similarity to ideal solution* (TOPSIS)” methods, Dinçer and Yüksel [24] assessed and ranked the renewable energy candidates based on specific criteria. With the use of the Pythagorean fuzzy “*vlsekriterijumska optimizacija I kompromisno resenje* (*VIKOR*)” model, Rani et al. [67] evaluated and ranked the candidate RESs in India. Motivated by reliability attributes, Aikhuele et al. [4] recommended a collective fuzzy decision support system for the assessment of RESs. Alkan and Albayrak [7] employed the MCDM methods to solve RES evaluations. Later, Ghenai et al. [32] presented an approach consisting of “*additive ratio assessment* (*ARAS*)” and “*step-wise weight assessment ratio analysis* (*SWARA*)” methods for RESs evaluation. Pan et al. [60] presented a hybrid interval type-2 fuzzy evidential reasoning model for choosing the most optimum RES candidate. With the use of triangular neutrosophic numbers, Abdel-Basset et al. [1] recommended an innovative evaluation model for RES selection. Ecer et al. [27] assessed and prioritized the RESs using a combinative distance-based assessment with an interval rough number. For the assessment of RES alternatives, Karatop et al. [40] put forward a collective framework by integrating “*FAHP*”, “*fuzzy failure mode and effect analysis *(*FEMEA*)” and “*evaluation based on distance from average solution *(*EDAS*)” techniques. Krishankumar et al. [45] designed a decision support system to prioritize the RES alternatives on “*q-rung orthopair fuzzy sets *(*q-ROFSs*)”. Their results concluded that solar and biomass energies are the most suitable alternatives for a given study in the Karnataka region.

### Fermatean fuzzy sets (FFSs)

Since the appearance of IFSs and PFSs, a tremendous amount of work has been done by researchers [26, 29]. Although, in many practical MCDM problems, there may be a case, where the DMEs give their view as (0.8, 0.7). Consequently, IFSs and PFSs are not capable of handling this case, since $0.8+0.7>1$ and ${0.8}^{2}+{0.7}^{2}>1$. To demonstrate this situation, Senapati & Yager [73] established the notion of FFS, represented by the MD and NMD, such that their cube sum is equal to or less than one. As a result, the FFSs act as a significant way to handle the MCDM process in an uncertain and complex environment and efficiently fill the gaps in IFS and PFS theories. Because of the ever-increasing complexity and widespread changes in the environment, a large number of researchers have shown remarkable interest in the MCDM problems with Fermatean fuzzy data. Based on Dombi operations, Aydemir & Gunduz [9] studied some Fermatean fuzzy Dombi AOs. In a recent study, Keshavarz-Ghorabaee et al. [43] and Mishra and Rani [53] presented the novel FFI based “*weighted aggregated sum product assessment *(*WASPAS*)” technique for green construction supplier assessment and healthcare waste disposal location selection, respectively. Inspired by the Hamacher operational laws, Hadi et al. [36] defined some Hamacher AOs under the FFS context and further utilized them to introduce a novel MCDM framework for cyclone disaster assessment. Deng and Wang [20] introduced an innovative Fermatean fuzzy MCDM technique by combining the Dempster–Shafer theory and Fermatean fuzzy entropy. Rani and Mishra [64] introduced a novel MULTIMOORA technique using the combination of Einstein operators and divergence measure within the FFS context and presented its application to the electric vehicle charging station selection problem. Furthermore, some studies have presented different decision-making approaches to enhance the investigation of the FFSs with AOs and information measures [5, 10, 34, 65]. Nevertheless, there is no study regarding the RES evaluation with Fermatean fuzzy data.

### Archimedean copula

Sklar [77] established the notion of “*copula*” which is a useful mathematical tool to aggregate probability distributions. Beliakov et al. [13] and Nelsen [58] put forward a methodical preface of copula and its relevance in information aggregation. Nather [57] utilized the copula to deal with probabilistic fuzzy information. Grabisch et al. [33] put forward several methods to construct aggregation functions, including copula. Bacigal et al. [11] considered aggregation functions preserving additive generators of “*Archimedean copulas*”. Tao et al. [78] termed copulas as functions that connect more than one marginal distribution to avoid information loss during the aggregation process. In accordance with the “*Archimedean Copula and probabilistic unbalanced linguistic term set*”, Han et al. [37] established a “*multi-criteria group decision-making* (*MCGDM*)” framework. Wu et al. [86] suggested a series of AOs based on “*Archimedean copula and co-copula*” under a hesitant fuzzy set context. Based on the combination of extended copulas, co-copulas and power average operators, Xu et al. [87] pioneered some copula power AOs on “*linguistic interval-valued intuitionistic fuzzy *(*LIVIFSs*)” and their applicability in MCGDM problems. Rong and Pei [69] studied some “*Archimedean copula and co-copula based interval-valued intuitionistic fuzzy generalized Bonferroni operators*”. In a study, Liu et al. [49] presented some “*q-rung orthopair fuzzy Banzhaf–Choquet-copula AOs*” and their applicability in MCGDM problems.

### Maclaurin symmetric mean (MSM)

The MSM operator [50, 61] can make the information aggregation process more realistic as it is capable of capturing the interrelationships among multi-input criteria via the variable parameters. Liu and Qin [48] pioneered several MSM operators within a linguistic IFS context. Teng et al. [80] introduced some power MSM operators based on “*Pythagorean fuzzy linguistic numbers*” and utilized for MCGDM problems. Wei and Lu [85] suggested MSM operators for PFSs and further studied their enviable characteristics. Based on “*Schweizer–Sklar operations*”, Wang and Liu [84] proposed some intuitionistic fuzzy Schweizer–Sklar MSM AOs and then applied them to MCGDM problems. Furthermore, Wang et al. [83] introduced a series of “*q-rung interval-valued orthopair fuzzy MSM operators*” with their relevance in group decision-making. In a study, Zhang [94] developed the concept of “*dual hesitant fuzzy MSM operator*” and its applicability. To aggregate the “*dual hesitant fuzzy soft numbers*”, Garg and Arora [31] studied some MSM AOs by means of t-norm operations. Liu et al. [47] investigated the MSM and partitioned Bonferroni mean within the context of IFSs and developed the partitioned MSM operator for intuitionistic fuzzy numbers.

### Similarity measure

The concept of “*similarity measure *(*SM*)” is a momentous and essential tool for quantifying the degree of closeness between any number of objects. Numerous scholars have worked on SMs in various fuzzy contexts and effectively used them to handle problems related to image processing, texture analysis, pattern identification, disease diagnosis, and decision-making, since the FSs were pioneered [62, 75, 76]. Recently, to evaluate the “*waste electrical and electronic equipment *(*WEEE*)” recycling partner, a similarity measure-based combined compromise solution model has been presented by Rani and Mishra [68]. For the sustainable biomass crop assessment, Mishra et al. [56] used the single-valued neutrosophic weighted aggregated sum product assessment model with the similarity measure. Rani and Mishra [68] and Mishra et al. [51] presented the “additive ratio assessment (ARAS)” method with the similarity measures on “interval‑valued intuitionistic fuzzy sets (IVIFSs)” to solve the low-carbon tourism strategy assessment. However, the notion of Fermatean fuzzy SM has been less investigated in the literature [10]. In this study, we develop some FF-SMs and discuss their elegant properties to obtain the criteria weight for solving MCDM problems.

### Complex proportional assessment (COPRAS) method

Literature consists of many MCDM approaches developed to solve complex selection problems that may arise daily. Essentially, a selection problem involves four main elements: alternatives, criteria, relative criteria weights, and measures the performance of the alternatives over preferred criteria. Multi-criteria decision analysis aims to select a desirable item from a set of possible choices, considering different criteria that may even conflict with each other. Zavadskas et al. [92] first proposed the COPRAS framework, which can be reasonably and effectively applied for information processing purposes. According to Darko and Liang [19], Dhiman and Deb [22], Dikshit-Ratnaparkhi et al. [23], COPRAS offers a suitable manner to effectively tackle the MCDM problems. The COPRAS method, which delivers more accurate information in comparison with different procedures for the evaluation of the benefits or cost criteria, is employed to assume both aspects of the criteria. In addition, COPRAS is able to delineate the ratios simultaneously to both ideal and worst solutions. Owing to its advantages, several researchers have employed the COPRAS approach for different purposes [16, 46, 54]. As MCDM problems have become increasingly complex and uncertain, different researchers have extended the conventional COPRAS approach to a variety of uncertain environments. For example, the COPRAS method has been discussed by Keshavarz-Ghorabaee et al. [42] in terms of its capacity for selecting optimum suppliers on “*interval type-2 fuzzy sets *(*IT2FSs*)”. In another project, the COPRAS approach with grey numbers was introduced by Bekar et al. [12] with the aim of evaluating the MCDM process. Zheng et al. [95] studied the COPRAS approach and its applications in the medical field on “*hesitant fuzzy linguistic term sets *(*HFLTSs*)”. Mishra et al. [55] suggested the COPRAS model to enlighten the correlative multiple criteria decision-making problems on “*hesitant fuzzy sets *(*HFSs*)”. Büyüközkan and Göçer [16] proposed a method that combined AHP and COPRAS models in a PFS context. Alipour et al. [6] evaluated fuel cell and hydrogen component suppliers using the Pythagorean fuzzy Entropy–SWARA–COPRAS method. To handle the cloud vendor assessment problem, Krishankumar et al. [44] extended the COPRAS technique under a probabilistic hesitant fuzzy set environment. For the first time, the present paper captures the combined advantages of FFS, Archimedean copula MSM and COPRAS methods, and introduces an innovative FFI-based MCDM framework to evaluate and rank the RES alternatives.

## Proposed similarity measures for FFSs

In this part, we first present the elementary ideas about the FFSs, Archimedean copula and Maclaurin symmetric mean operator. Furthermore, we introduce two innovative Fermatean fuzzy similarity measures to quantify the degree of similarity between FFSs.

### Basic concepts

Let us give a brief review of some essential concepts that are concerned with the present study.

#### **Definition 3.1**

[73] A FFS *F* on a discourse set $\Upsilon$ is defined mathematically as $F = \left\{ {\left. {\left\langle {y_{i} ,\,F\left( {\mu_{F} (y_{i} ),\,\nu_{F} (y_{i} )} \right)} \right\rangle } \right|\,y_{i} \, \in \,\Upsilon } \right\},$ where $\mu_{F} ,\,\nu_{F} \,:\,\Upsilon \, \to \,\left[ {0,\,1} \right]$ symbolize the MD and NMD of the object $y_{i} \, \in \,\Upsilon$ to *F*, respectively, with the condition $0\, \le \,\left( {\mu_{F} \left( {y_{i} } \right)} \right)^{3} \, + \,\left( {\nu_{F} \left( {y_{i} } \right)} \right)^{3} \, \le \,1.$ The hesitancy function is defined by $\pi_{F} \left( {y_{i} } \right) = \,\sqrt[3]{{1\, - \,\mu_{F}^{3} \left( {y_{i} } \right) - \,\nu_{F}^{3} \left( {y_{i} } \right)}},\,\,\forall \,y_{i} \, \in \,\Upsilon .$ For simplicity, Senapati and Yager [73] defined by the “*Fermatean fuzzy number *(*FFN*)” by $\alpha = \,F\left( {\mu_{\alpha } ,\,\nu_{\alpha } } \right)$ which satisfies $\mu_{\alpha } ,\,\nu_{\alpha } \, \in \,\left[ {0,\,1} \right]$ and $0\, \le \,\mu_{\alpha }^{3} \, + \,\nu_{\alpha }^{3} \, \le \,1.$

#### **Definition 3.2**

[73] Consider a FFN $\alpha = \,F\left( {\mu_{\alpha } ,\,\nu_{\alpha } } \right).$ Then the score value and accuracy value of $\alpha$ are defined by

1 $$ {\mathbb{S}}\left( \alpha \right) = \left( {\mu_{\alpha } } \right)^{3} - \left( {\nu_{\alpha } } \right)^{3} \quad {\text{and}}\quad \hbar \left( \alpha \right) = \left( {\mu_{\alpha } } \right)^{3} + \left( {\nu_{\alpha } } \right)^{3} , $$

where $${\mathbb{S}}\left( \alpha \right) \in \left[ { - 1,\,1} \right]$$ and $$\hbar \left( \alpha \right) \in \left[ {0,1} \right].$$

As the score function ${\mathbb{S}}\left( \alpha \right) \in \left[ { - 1,\,1} \right],$ therefore, the normalized score value for a FFN $\alpha = \,F\left( {\mu_{\alpha } ,\,\nu_{\alpha } } \right)$ is presented as

2 $$ {\mathbb{S}}^{ * } \left( \alpha \right)\, = \,\frac{1}{2}\left( {{\mathbb{S}}\left( \alpha \right)\, + \,1} \right), $$

where $${\mathbb{S}}^{*} \left( \alpha \right) \in \left[ {0,1} \right].$$

For any two FFNs $\alpha_{1} \, = \,F\left( {\mu_{{\alpha_{1} }} ,\,\nu_{{\alpha_{1} }} } \right)$ and $\alpha_{2} \, = \,F\left( {\mu_{{\alpha_{2} }} ,\,\nu_{{\alpha_{2} }} } \right),$ we have.

(i) If $${\mathbb{S}}^{*} \left( {\alpha_{1} } \right) > {\mathbb{S}}^{*} \left( {\alpha_{2} } \right),$$ then $$\alpha_{1} > \,\alpha_{2} ,$$
(ii) If ${\mathbb{S}}^{*} \left( {\alpha_{1} } \right) = {\mathbb{S}}^{*} \left( {\alpha_{2} } \right),$ then
  - if $$\hbar \left( {\alpha_{1} } \right)\, < \hbar \left( {\alpha_{2} } \right),$$ then $$\alpha_{1} < \,\alpha_{2} ;$$
  - if $$\hbar \left( {\alpha_{1} } \right) > \hbar \left( {\alpha_{2} } \right),$$ then $$\alpha_{1} > \,\alpha_{2} ;$$
  - if $$\hbar \left( {\alpha_{1} } \right) = \hbar \left( {\alpha_{2} } \right),$$ then $$\alpha_{1} = \,\alpha_{2} .$$

#### **Definition 3.3**

[73] Let $$\alpha \, = \,F\left( {\mu_{\alpha } ,\,\nu_{\alpha } } \right),$$$$\alpha_{1} \, = \,F\left( {\mu_{{\alpha_{1} }} ,\,\nu_{{\alpha_{1} }} } \right),$$ $$\alpha_{2} \, = \,F\left( {\mu_{{\alpha_{2} }} ,\,\nu_{{\alpha_{2} }} } \right) \in FFNs\left( \Upsilon \right).$$ Then, the operations on FFNs are defined as

(i) $$\alpha^{c} \, = \,F\left( {\nu_{\alpha } ,\,\mu_{\alpha } } \right);$$
(ii) $$\alpha_{1} \, \cap \,\alpha_{2} \, = \,F\left( {\min \left\{ {\mu_{{\alpha_{1} }} \,,\,\mu_{{\alpha_{2} }} } \right\},\,\,\,\max \left\{ {\nu_{{\alpha_{1} }} \,,\,\nu_{{\alpha_{2} }} } \right\}} \right);$$
(iii) $$\alpha_{1} \, \cup \,\alpha_{2} \, = \,F\left( {\max \left\{ {\mu_{{\alpha_{1} }} \,,\,\mu_{{\alpha_{2} }} } \right\},\,\,\,\min \left\{ {\nu_{{\alpha_{1} }} \,,\,\nu_{{\alpha_{2} }} } \right\}} \right);$$
(iv) $$\alpha_{1} \, \oplus \,\alpha_{2} \, = \,F\left( {\sqrt[3]{{\mu_{{\alpha_{1} }}^{3} \, + \,\mu_{{\alpha_{2} }}^{3} \, - \,\mu_{{\alpha_{1} }}^{3} \,\mu_{{\alpha_{2} }}^{3} }},\,\nu_{{\alpha_{1} }} \,\nu_{{\alpha_{2} }} } \right);$$
(v) $$\alpha_{1} \, \otimes \,\alpha_{2} \, = \,F\left( {\mu_{{\alpha_{1} }} \,\mu_{{\alpha_{2} }} ,\,\sqrt[3]{{\nu_{{\alpha_{1} }}^{3} \, + \,\nu_{{\alpha_{2} }}^{3} \, - \,\nu_{{\alpha_{1} }}^{3} \,\nu_{{\alpha_{2} }}^{3} }}} \right);$$
(vi) $$\begin{aligned}\alpha_{1} \,\Theta \,\alpha_{2} \,
=
\,\left\{ \begin{array}{l} F\left(
{\sqrt[3]{{\frac{{\mu_{{\alpha_{1} }}^{3} - \mu_{{\alpha_{2} }}^{3}
}}{{1 - \mu_{{\alpha_{2} }}^{3} }}}},\,\frac{{\nu_{{\alpha_{1} }}
\,}}{{\nu_{{\alpha_{2} }} }}\,} \right),\\
\quad\mu_{{\alpha_{1} }} \ge
\,\mu_{{\alpha_{2} }} ,\,\nu_{{\alpha_{1} }} \le \min \left\{
{\nu_{{\alpha_{2} }} ,\,\frac{{\nu_{{\alpha_{2} }}
\,\pi_{{\alpha_{1} }} }}{{\pi_{{\alpha_{2} }} }}} \right\} \hfill \\
F\left( {0,1\,}
\right),\,\,\,\,\,\,\,\,\,\,\,\,\,\,\,\,\,\,\,\,\,\,\,\,\,\,{\text{otherwise}}\,\,
\hfill \\ \end{array}
\right.\end{aligned}$$
(vii) $$\lambda \,\alpha \, = \,F\left( {\sqrt[3]{{1 - \left( {1 - \,\mu_{\alpha }^{3} } \right)^{\lambda } }}\,,\,\left( {\nu_{\alpha } } \right)^{\lambda } } \right),\,\,\lambda > \,0;$$
(viii) $$\alpha^{\lambda } \, = \,F\left( {\left( {\mu_{\alpha } } \right)^{\lambda } ,\,\sqrt[3]{{1 - \left( {1 - \,\nu_{\alpha }^{3} } \right)^{\lambda } }}} \right),\,\,\lambda \, > \,0.$$

#### **Definition 3.4**

[77] A copula is a mapping ${\mathbb{C}}:[0,1] \times [0,1] \to [0,1]$ satisfying the subsequent conditions:

(i) $${\mathbb{C}}(t,0) = {\mathbb{C}}(0,t) = 0,\,{\mathbb{C}}(t,1) = {\mathbb{C}}(1,t) = t\,\,\forall \,t\, \in [0,1]$$
(ii) $${\mathbb{C}}(t_{1} ,s_{1} ) + {\mathbb{C}}(t_{2} ,s_{2} ) - {\mathbb{C}}(t_{2} ,s_{1} ) - {\mathbb{C}}(t_{1} ,s_{2} ) \ge 0,\,\,{\text{for}}\,\,t_{1} ,s_{1} ,t_{2} ,s_{2} \in [0,1]\,\,{\text{with}}\,\,t_{1} \le t_{2} \,\,{\text{and}}\,\,\,s_{1} \le s_{2} .$$

#### **Definition 3.5**

[58] An Archimedean copula is a mapping $\varphi :[0,1] \times [0,1] \to [0,1]$ presented by $\varphi (t,s) = \psi (\eta (t) + \eta (s)),$ where $\eta :[0,1] \to [0,\infty )$ is a strictly decreasing mapping and $\psi :[0,\infty ) \to [0,1]$ is given as

$$ \psi (t) = \left\{ {\begin{array}{*{20}c} {\eta^{ - 1} (t),\,\,\,t \in [0,\,\eta (0)],} \\ {0,\,\,\,t \in [\eta (0),\,\,\infty ].} \\ \end{array} } \right. $$

An Archimedean copula is termed as a strict Archimedean copula if $\varphi$ is strictly increasing on $[0,1] \times [0,1]$ and $\psi$ becomes identical with $\eta .$ In such a scenario $\varphi (t,s) = \eta^{ - 1} (\eta (t) + \eta (s))$.

#### **Definition 3.6**

[50, 61] The MSM operator of the non-negative real numbers $\xi_{1} ,\xi_{2} ,...,\xi_{n}$ is defined by

$$ \begin{aligned} & {\text{MSM}}^{(r)} (\xi_{1} ,\xi_{2} , \ldots ,\xi_{n} ) \\ &\quad = \left( {\frac{1}{{{}^{n}c_{r} }}\sum\limits_{{1 \le p_{1} < p_{2} < \cdots < p_{r} \le n}} {\left( {\prod\limits_{j = 1}^{r} {\xi_{{p_{j} }} } } \right)} } \right)^{\frac{1}{r}} , \end{aligned} $$

where *r* is a parameter, ${}^{n}c_{r}$ stands for binomial coefficient and $(p_{1} ,p_{2} ,...,p_{r} )$ denotes a *r*-tuple combination of $(1,2, \ldots ,n)$.

### Novel Fermatean fuzzy similarity measures

Motivated by this idea, we develop two new SMs for FFSs in this section and then apply them to the Fermatean fuzzy COPRAS method for evaluating the criteria weights. Based on Liu et al. [49] measure, we develop the following SM for FFSs:

#### **Definition 3.7**

Let $F,H,\,I\, \in \,FFSs\left( \Upsilon \right).$ A Fermatean fuzzy SM $S\,:\,FFS(\Upsilon )\, \times \,FFS(\Upsilon )\, \to {\mathbb{R}}$ is a real-valued function which satisfies the following axioms:

1. $$0\, \le \,S\left( {F,\,H} \right)\, \le 1,$$
2. $$S\left( {F,\,H} \right)\, = \,S\left( {H,\,F} \right),$$
3. $$S\left( {F,\,H} \right)\, = \,1$$ iff $$F = \,H\,,$$
4. $S\left( {F,\,F^{c} } \right)\, = 0$ iff $F$ is a crisp set,
5. If $F \subseteq \,H\, \subseteq \,I,$ then $S\left( {F,\,I} \right)\, \le \,S\left( {F,\,H} \right)$ and $S\left( {F,\,I} \right)\, \le \,S\left( {H,\,I} \right).$

Based on Liu et al. [49], we first develop the axiomatic definition of Fermatean fuzzy similarity measure as follows:

3 $$ S_{1} \left( {F,H} \right) = \frac{{\sum\nolimits_{i = 1}^{n} {\left( {\min \left\{ {\mu_{F}^{3} \left( {y_{i} } \right),\,\mu_{H}^{3} \left( {y_{i} } \right)} \right\} + \min \left\{ {1 - \nu_{F}^{3} \left( {y_{i} } \right),\,1 - \nu_{H}^{3} \left( {y_{i} } \right)} \right\}} \right)} }}{{\sum\nolimits_{i = 1}^{n} {\left( {\max \left\{ {\mu_{F}^{3} \left( {y_{i} } \right),\,\mu_{H}^{3} \left( {y_{i} } \right)} \right\} + \max \left\{ {1 - \nu_{F}^{3} \left( {y_{i} } \right),\,1 - \nu_{H}^{3} \left( {y_{i} } \right)} \right\}} \right)} }},\quad \forall \,y_{i} \, \in \,\Upsilon . $$

#### **Theorem 3.1**

*The function* $S_{1} \left( {F,\,H} \right),$ *given by* (3), *is a SM for FFSs*.

#### *Proof*

(a_1_) and (a_2_). Both are obvious from Definition 3.7.

(a_3_). Let $F,\,H \in FFSs\left( \Upsilon \right)$ and $F = H,$ that is, $\mu_{F} \left( {y_{i} } \right) = \mu_{H} \left( {y_{i} } \right)$ and $\nu_{F} \left( {y_{i} } \right) = \nu_{H} \left( {y_{i} } \right)\,.$ Therefore, from Eq. (3), we obtain $S_{1} \left( {F,H} \right) = 1.$

Again, let $S_{1} \left( {F,H} \right) = 1.$ Then, from Eq. (3), we obtain

4 $$ \begin{aligned} & \sum\limits_{i = 1}^{n}\left[ \min \left\{ {\mu_{F}^{3} \left( {y_{i} }\right),\mu_{H}^{3} \left( {y_{i} } \right)} \right\}\right.\\&\left.\qquad + \min \left\{{\left( {1 - \nu_{F}^{3} \left( {y_{i} } \right)} \right),\left( {1- \nu_{H}^{3} \left( {y_{i} } \right)} \right)} \right\} \right]\\ & \quad = \sum\limits_{i = 1}^{n} \left[ \max \left\{{\mu_{F}^{3} \left( {y_{i} } \right),\,\mu_{H}^{3} \left( {y_{i} }\right)} \right\} \right.\\&\qquad\left.+ \max \left\{ {\left( {1 - \nu_{F}^{3} \left({y_{i} } \right)} \right),\left( {1 - \nu_{H}^{3} \left( {y_{i} }\right)} \right)} \right\} \right] . \\ \end{aligned}$$

As $$\forall \,\,y_{i} \in
\,Y,$$$$\min \left\{
{\mu_{F}^{3} \left( {y_{i} } \right),\mu_{H}^{3} \left( {y_{i} }
\right)} \right\} \le \max \left\{ \mu_{F}^{3} \left( {y_{i} }
\right), \mu_{H}^{3} \left( {y_{i} } \right)
\right\}\,,$$ and $$\min \left\{ {\left( {1 - \nu_{F}^{3} \left( {y_{i} } \right)} \right),\left( {1 - \nu_{H}^{3} \left( {y_{i} } \right)} \right)} \right\} \le \max \left\{ {\left( {1 - \nu_{F}^{3} \left( {y_{i} } \right)} \right),\left( {1 - \nu_{H}^{3} \left( {y_{i} } \right)} \right)} \right\}.$$ Therefore,$$S_{1} \left( {F,\,H} \right) = 1\,$$ will be true when $$\mu_{F} \left( {y_{i} } \right) = \mu_{H} \left( {y_{i} } \right)\,$$ and $$\left( {1 - \nu_{F} \left( {y_{i} } \right)} \right) = \,\left( {1 - \nu_{H} \left( {y_{i} } \right)} \right)\,.$$ It implies that $$F = \,H.$$

(a_4_). It is clear from Eq. (3).

(a_5_). Let $F,\,H,\,I\, \in \,FFSs\left( \Upsilon \right)$ and $F \subseteq H \subseteq I,$ i.e., $\mu_{F} \left( {y_{i} } \right) \le \mu_{H} \left( {y_{i} } \right)\, \le \mu_{I} \left( {y_{i} } \right)\,$ and $\nu_{F} \left( {y_{i} } \right) \ge \nu_{H} \left( {y_{i} } \right) \ge \nu_{I} \left( {y_{i} } \right)\,\,,$ $\forall \,\,y_{i} \, \in \,\Upsilon .$ Also, $\left( {1 - \nu_{F}^{3} \left( {y_{i} } \right)} \right) \le \left( {1 - \nu_{H}^{3} \left( {y_{i} } \right)} \right)\, \le \left( {1 - \nu_{I}^{3} \left( {y_{i} } \right)} \right).$

Now,

$$ \begin{aligned}&S_{1} \left( {F,\,I} \right) \\
&\,\,=
\frac{{\sum\nolimits_{i = 1}^{n} \!{\left[\! {\min \!\left\{ {\mu_{F}^{3}
\left( {y_{i} } \right),\mu_{I}^{3} \!\left( {y_{i} } \right)}
\right\} \!+\! \min \!\left\{\! {\left( {1 - \nu_{F}^{3} \left( {y_{i} }
\right)} \right),\!\left( {1\! -\! \nu_{I}^{3} \left( {y_{i} } \right)}
\right)} \!\right\}} \!\right]} }}{{\sum\nolimits_{i = 1}^{n} \!{\left[\!
{\max \!\left\{ \!{\mu_{F}^{3}\! \left( {y_{i} } \right),\mu_{I}^{3}
\left( {y_{i} } \right)}\! \right\}\! +\! \max \!\left\{ \!{\left( {1\! -\!
\nu_{F}^{3} \left( {y_{i} } \right)} \!\right),\!\left( {1 \!-\! \nu_{I}^{3}
\left( {y_{i} } \right)}\! \right)}\! \right\}} \!\right]} }},\end{aligned}
$$

implies that

5 $$ S_{1} \left( {F,I} \right) = \frac{{\sum\nolimits_{i = 1}^{n} {\left[ {\mu_{F}^{3} \left( {y_{i} } \right) + \left( {1 - \nu_{F}^{3} \left( {y_{i} } \right)} \right)} \right]} }}{{\sum\nolimits_{i = 1}^{n} {\left[ {\mu_{I}^{3} \left( {y_{i} } \right) + \left( {1 - \nu_{I}^{3} \left( {y_{i} } \right)} \right)} \right]} }}. $$

Similarly,

6 $$ S_{1} \left( {F,H} \right) = \frac{{\sum\nolimits_{i = 1}^{n} {\left[ {\mu_{F}^{3} \left( {y_{i} } \right) + \left( {1 - \nu_{F}^{3} \left( {y_{i} } \right)} \right)} \right]} }}{{\sum\nolimits_{i = 1}^{n} {\left[ {\mu_{H}^{3} \left( {y_{i} } \right) + \left( {1 - \nu_{H}^{3} \left( {y_{i} } \right)} \right)} \right]} }}. $$

Thus, from Eqs. (5) and (6), we obtain

$$ S_{1} \left( {F,\,H} \right) \ge S_{1} \left( {F,\,I} \right). $$

Similarly, we can prove that $S_{1} \left( {H,\,I} \right) \ge S_{1} \left( {F,\,I} \right)\,.$ [Proved].

After that, another Fermatean fuzzy SM is proposed using the combination of $S_{1} \left( {F,\,H} \right)$ and a lattice. In general, “*a lattice of a nonempty set is a hierarchical structure prepared by a partial order such that for every two objects in the lattice, there exists a lub* (*supremum*) *and a glb* (*infimum*)”. Similarity between two objects in a lattice is typically calculated using information from their supremum and infimum.

Utilizing the FFSs as a lattice concept and the subset connection given in Eq. (3) as a partial order, a lattice can be generated. For two FFSs, the supremum and infimum, can be accessed from the union and intersection, respectively. Consequently, the new similarity measure for FFSs is defined as

7 $$ \begin{aligned}&S_{2} \left( {F,H} \right) = \sqrt {S_{1}
\left( {F,C_{FH} } \right) \times S_{1} \left( {H,C_{FH} } \right)},\\
&\qquad\qquad\qquad {\text{where }}C_{FH} = F \cup H.\end{aligned}
$$

#### **Theorem 3.2**

*The function*, *shown in* Eq. (7), *is a valid SM for FFSs*.

#### *Proof*

(a_1_) and (a_2_). Both are obvious.

(a_3_). Let $$F,\,H \in FFSs\left( Y \right)$$ and $$F = \,H.$$ Since $$C_{FH} = F \cup H,$$ therefore, $$F = H = C_{FH}$$ and $$S_{1} \left( {F,\,H} \right)$$ satisfies the property (a_3_). Hence, $$S_{2} \left( {F,\,H} \right) = 1.$$ Again, let $$S_{2} \left( {F,\,H} \right) = 1.$$ This implies $$S_{1} (F,C_{FH} ) =$$$$S_{1} (H,C_{FH} ) = 1,$$ when $$C_{FH} = F \cup H$$ and $$S_{1} (F,\,H)$$ satisfies (a_3_). Hence, $$F = \,H = C_{FH} .$$

(a_4_). It is clear from Eq. (7).

(a_5_). Let $F,\,H,\,I \in FFSs\left( \Upsilon \right)$ and $F \subseteq H \subseteq I.$ Then, $F \cup H = H,$ $F \cup I = I$ and $H \cup I = I.$

Now, $S_{2} (F,\,I) = \sqrt {S_{1} \left( {F,C_{FI} } \right) \times S_{1} \left( {I,C_{FI} } \right)} ,$ which implies that $S_{2} \left( {F,\,I} \right) = \sqrt {S_{1} \left( {F,\,I} \right) \times S_{1} \left( {I,\,I} \right)} .$ Thus, $S_{2} \left( {F,\,I} \right) = \sqrt {S_{1} \left( {F,I} \right)} .$ Similarly, we can verify that $S_{2} (F,\,H) = \sqrt {S_{1} \left( {F,\,H} \right)} .$ As $S_{1} (F,\,I)$ holds (a_5_), that is, $S_{1} \left( {F,\,H} \right) \ge S_{1} \left( {F,I} \right),$ therefore,$S_{2} \left( {F,\,H} \right) \ge S_{2} \left( {F,\,I} \right).$ Similarly, $S_{2} \left( {H,\,I} \right) \ge \,S_{2} \left( {F,\,I} \right).$ [Proved].

## Proposed Fermatean fuzzy Archimedean copula MSM operator

This section presents the Fermatean fuzzy Archimedean copula weighted MSM (FFACWMSM) operator based on the Archimedean copula and the MSM operator, and then the axioms of this new operator are verified and some particular cases are conferred. For this, we first present the Archimedean copula-based operations between FFNs.

### **Definition 4.1**

Let $\alpha_{1} = F(\mu_{{\alpha_{1} }} ,\nu_{{\alpha_{1} }} )$ and $\alpha_{2} = F(\mu_{{\alpha_{2} }} ,\nu_{{\alpha_{2} }} )$ be two FFNs and $\lambda > 0$. Then the Archimedean Copula-based operations between the FFNs are defined as

1. $$\alpha_{1} \,\tilde{ \oplus }\,\alpha_{2} = F\left( \sqrt[3]{{1 - \varphi^{ - 1} (\varphi (1 - \mu_{{\alpha_{1} }}^{3} ) + \varphi (1 - \mu_{{\alpha_{2} }}^{3} ))}},\right.$$
  $$\qquad\qquad\qquad\left.\sqrt[3]{{\varphi^{ - 1} (\varphi (\nu_{{\alpha_{1} }}^{3} ) + \varphi (\nu_{{\alpha_{2} }}^{3} ))}} \right)$$
2. $$\alpha_{1} \tilde{\otimes }\alpha_{2} = F\left( \sqrt[3]{{\varphi^{ - 1} (\varphi (\mu_{{\alpha_{1} }}^{3} ) + \varphi (\mu_{{\alpha_{2} }}^{3} ))}}, \right. \break\left. \sqrt[3]{{1 - \varphi^{ - 1} (\varphi (1 - \nu_{{\alpha_{1} }}^{3} ) + \varphi (1 - \nu_{{\alpha_{2} }}^{3} ))}} \right)$$
3. $$\lambda \alpha_{1} = F\left( {\sqrt[3]{{1 - \varphi^{ - 1} (\lambda \varphi (1 - \mu_{{\alpha_{1} }}^{3} ))}},\,\sqrt[3]{{\varphi^{ - 1} (\lambda \varphi (\nu_{{\alpha_{1} }}^{3} ))}}} \right)$$
4. $$\alpha_{1}^{\lambda } = F\left( {\sqrt[3]{{\varphi^{ - 1} (\lambda \varphi (\mu_{{\alpha_{1} }}^{3} ))}},\,\sqrt[3]{{1 - \varphi^{ - 1} (\lambda \varphi (1 - \nu_{{\alpha_{1} }}^{3} ))}}} \right).$$

In accordance with Definition 4.1, we develop the following theorem:

### **Theorem 4.1**

*Let* $\alpha_{1} = F(\mu_{{\alpha_{1} }} ,\nu_{{\alpha_{1} }} )$ *and* $\alpha_{2} = F(\mu_{{\alpha_{2} }} ,\nu_{{\alpha_{2} }} )$ *be two FFNs and* $\lambda ,\lambda_{1} ,\lambda_{2} > 0$. *Then*, *we have*

(i) $$\alpha_{1} \,\tilde{ \oplus }\,\alpha_{2} = \alpha_{2} \,\tilde{ \oplus }\,\alpha_{1}$$
(ii) $$\alpha_{1} \tilde{ \otimes }\alpha_{2} = \alpha_{2} \tilde{ \otimes }\alpha_{1}$$
(iii) $$\lambda (\alpha_{1} \,\tilde{ \oplus }\,\alpha_{2} ) = \lambda \alpha_{1} \,\tilde{ \oplus }\,\lambda \alpha_{2}$$
(iv) $$(\alpha_{1} \tilde{ \otimes }\alpha_{2} )^{\lambda } = \alpha_{1}^{\lambda } \tilde{ \otimes }\alpha_{2}^{\lambda }$$
(v) $$(\lambda_{1} + \lambda_{2} )\alpha_{1} = \lambda_{1} \alpha_{1} \,\tilde{ \oplus }\,\lambda_{2} \alpha_{1}$$
(vi) $$\alpha_{1}^{{\lambda_{1} + \lambda_{2} }} = \alpha_{1}^{{\lambda_{1} }} \tilde{ \otimes }\alpha_{1}^{{\lambda_{2} }} .$$

### *Proof*

(i)–(ii). Both are straightforward.

(iii). We have

$$ \begin{aligned} &
\lambda (\alpha_{1} \,\tilde{ \oplus }\,\alpha_{2} ) \\ & \quad =\!
F\left( \sqrt[3]{{1 \!-\! \varphi^{ - 1} (\lambda \varphi (1 \!-\! (1 \!-\!
\varphi^{ - 1} (\varphi (1 \!-\! \mu_{{\alpha_{1} }}^{3} ) + \varphi (1
\!-\! \mu_{{\alpha_{2} }}^{3} )))))}},\right.\\
&\qquad\left.\sqrt[3]{{\varphi^{ - 1}
(\lambda \varphi (\varphi^{ - 1} (\varphi (\nu_{{\alpha_{1} }}^{3} )
+ \varphi (\nu_{{\alpha_{2} }}^{3} )))))}} \right) \\ & \quad =
F\left( \sqrt[3]{{1 - \varphi^{ - 1} (\lambda \varphi (1 -
\mu_{{\alpha_{1} }}^{3} ) + \lambda \varphi (1 - \mu_{{\alpha_{2}
}}^{3} ))}},\right.\\
&\qquad\left.\sqrt[3]{{\varphi^{ - 1} (\lambda \varphi
(\nu_{{\alpha_{1} }}^{3} ) + \varphi (\lambda \nu_{{\alpha_{2}
}}^{3} ))}} \right). \\ \end{aligned}
$$

On the other hand

$$ \begin{aligned} &
\lambda \alpha_{1} \,\tilde{ \oplus }\,\lambda \alpha_{2} \\ & \quad =
F\left( \sqrt[3]{{1 - \varphi^{ - 1} (\lambda \varphi (1 -
\mu_{{\alpha_{1} }}^{3} ))}},\,\sqrt[3]{{\varphi^{ - 1} (\lambda
\varphi (\nu_{{\alpha_{1} }}^{3} ))}} \right)\\
&\qquad\,\tilde{ \oplus }\,F\left( {\sqrt[3]{{1 - \varphi^{ - 1} (\lambda \varphi (1 -
\mu_{{\alpha_{2} }}^{3} ))}},\,\sqrt[3]{{\varphi^{ - 1} (\lambda
\varphi (\nu_{{\alpha_{2} }}^{3} ))}}} \right) \\ & \quad = \!F\left(\!
{\sqrt[3]{{1\! - \!\varphi^{ - 1}\! (\varphi \!(1\! -\! (1\! -\! \varphi^{ - 1}
\!(\lambda \varphi (1 \!-\! \mu_{{\alpha_{1} }}^{3} )))) \!+\! \varphi (1 \!-\! (1
\!-\! \varphi^{ - 1} (\lambda \varphi (1 \!-\! \mu_{{\alpha_{2} }}^{3}\!
)))))}}} \right., \\ & \quad \quad \left. {\sqrt[3]{{\varphi^{ - 1}
(\varphi \!(\varphi^{ - 1} (\lambda \varphi\! (\nu_{{\alpha_{1} }}^{3}
))) + \varphi (\varphi^{ - 1} (\lambda \varphi (\nu_{{\alpha_{2}
}}^{3} ))))}}} \right) \\ & \quad = F\left( \sqrt[3]{{1 - \varphi^{
- 1} (\lambda \varphi (1 - \mu_{{\alpha_{1} }}^{3} ) + \lambda
\varphi (1 - \mu_{{\alpha_{2} }}^{3} ))}},\right.\\
&\qquad\left.\sqrt[3]{{\varphi^{ - 1}
(\lambda \varphi (\nu_{{\alpha_{1} }}^{3} ) + \varphi (\lambda
\nu_{{\alpha_{2} }}^{3} ))}} \right). \\ \end{aligned}
$$

This implies that $\lambda (\alpha_{1} \,\tilde{ \oplus }\,\alpha_{2} ) = \lambda \alpha_{1} \,\tilde{ \oplus }\,\lambda \alpha_{2}$.

Proof of (iv)–(vi) are similar to proof of (iii). Hence, we omitted the proof.

### Definition 4.2

Let $\alpha_{j} = F(\mu_{{\alpha_{j} }} ,\nu_{{\alpha_{j} }} )\,\,(j \in \Upsilon_{n} )$ be a collection of FFNs. Then an Archimedean copula based weighted MSM operator on FFNs is denoted by ${\text{FFACWMSM}}_{{{\text{AC}}}}^{(r)}$ and is defined by

8 $$\begin{aligned} &{\text{FFACWMSM}}_{{{\text{AC}}}}^{(r)}
(\alpha_{1} ,\alpha_{2} , \ldots ,\alpha_{n} )\\
&\quad = \left(
{\frac{1}{{{}^{n}c_{r} }}\mathop {\,\tilde{ \oplus }\,}\limits_{{1 \le
p_{1} < p_{2} < \cdots < p_{r} \le n}} \left( {\tilde{ \otimes }_{i
= 1}^{r} w_{{p_{i} }} \alpha_{{p_{i} }} } \right)}
\right)^{\frac{1}{r}} ,\end{aligned} $$

where ${}^{n}c_{r}$ stands for binomial coefficient, $(p_{1} ,p_{2} , \ldots ,p_{r} )$ denotes a *r*-tuple combination of $(1,2, \ldots ,n)$, and $w_{{p_{i} }}$ is the weight of $\alpha_{{p_{i} }} \,(i \in \Upsilon_{r} )$.

Based on Definitions 4.1 and 4.2, the following theorem is obtained:

### **Theorem 4.2**

*Let* $\alpha_{j} = F(\mu_{{\alpha_{j} }} ,\nu_{{\alpha_{j} }} )\,\,(j \in \Upsilon_{n} )$ *be a set of FFNs*. *Then*, *we have*

9 $$ \begin{aligned} & {\text{FFACWMSM}}_{{{\text{AC}}}}^{(r)} (\alpha_{1} ,\alpha_{2} , \ldots ,\alpha_{n} ) \\ & \quad = F\left( {\sqrt[3]{{\varphi^{ - 1} \left( {\frac{1}{r}\varphi \left( {1 - \varphi^{ - 1} \left( {\frac{1}{{{}^{n}c_{r} }}\sum\limits_{{1 \le p_{1} < p_{2} < \cdots < p_{r} \le n}} {\varphi \left( {1 - \varphi^{ - 1} \left( {\sum\limits_{i = 1}^{r} {\varphi (1 - \varphi^{ - 1} (w_{{p_{i} }} \varphi (1 - \mu_{{\alpha_{{p_{i} }} }}^{3} )))} } \right)} \right)} } \right)} \right)} \right)}}} \right., \\ & \quad \quad \left. {\sqrt[3]{{1 - \varphi^{ - 1} \left( {\frac{1}{r}\varphi \left( {1 - \varphi^{ - 1} \left( {\frac{1}{{{}^{n}c_{r} }}\sum\limits_{{1 \le p_{1} < p_{2} < \cdots < p_{r} \le n}} {\varphi \left( {1 - \varphi^{ - 1} \left( {\sum\limits_{i = 1}^{r} {\varphi (1 - \varphi^{ - 1} (w_{{p_{i} }} \varphi (\nu_{{\alpha_{{p_{i} }} }}^{3} )))} } \right)} \right)} } \right)} \right)} \right)}}} \right) \\ \end{aligned} $$

*where* ${}^{n}c_{r}$ *stands for binomial coefficient*, $(p_{1} ,p_{2} , \ldots ,p_{r} )$ *denotes a* *r*-*tuple combination of* $(1,2, \ldots ,n)$, *and* $w_{{p_{i} }}$ *is the weight of* $\alpha_{{p_{i} }} \,(i \in \Upsilon_{r} )$.

### *Proof*

By Definition 4.1, we have

$$ w_{{p_{i} }} \alpha_{{p_{i} }} = F\left( {\sqrt[3]{{1 - \varphi^{ - 1} (w_{{p_{i} }} \varphi (1 - \mu_{{\alpha_{{p_{i} }} }}^{3} ))}}} \right.,\,\left. {\sqrt[3]{{\varphi^{ - 1} (w_{{p_{i} }} \varphi (\nu_{{\alpha_{{p_{i} }} }}^{3} ))}}} \right). $$

Therefore

$$ \,\tilde{ \otimes }_{i = 1}^{r} w_{{p_{i} }} \alpha_{{p_{i} }} = F\left( {\sqrt[3]{{\varphi^{ - 1} \left( {\sum\limits_{i = 1}^{r} {\varphi (1 - \varphi^{ - 1} (w_{{p_{i} }} \varphi (1 - \mu_{{\alpha_{{p_{i} }} }}^{3} )))} } \right)}}} \right.,\,\,\left. {\sqrt[3]{{1 - \varphi^{ - 1} \left( {\sum\limits_{i = 1}^{r} {\varphi (1 - \varphi^{ - 1} (w_{{p_{i} }} \varphi (\nu_{{\alpha_{{p_{i} }} }}^{3} )))} } \right)}}} \right). $$

Now

$$ \begin{aligned} & \mathop {\,\tilde{ \oplus }\,}\limits_{{1 \le p_{1} < p_{2} < \cdots < p_{r} \le n}} \left( {\tilde{ \otimes }_{i = 1}^{r} w_{{p_{i} }} \alpha_{{p_{i} }} } \right) \\ & \quad = F\left( {\sqrt[3]{{1 - \varphi^{ - 1} \left( {\sum\limits_{{1 \le p_{1} < p_{2} < \cdots < p_{r} \le n}} {\varphi \left( {1 - \varphi^{ - 1} \left( {\sum\limits_{i = 1}^{r} {\varphi (1 - \varphi^{ - 1} (w_{{p_{i} }} \varphi (1 - \mu_{{\alpha_{{p_{i} }} }}^{3} )))} } \right)} \right)} } \right)}}} \right., \\ & \quad \quad \left. {\sqrt[3]{{\varphi^{ - 1} \left( {\sum\limits_{{1 \le p_{1} < p_{2} < \cdots < p_{r} \le n}} {\varphi \left( {1 - \varphi^{ - 1} \left( {\sum\limits_{i = 1}^{r} {\varphi (1 - \varphi^{ - 1} (w_{{p_{i} }} \varphi (\nu_{{\alpha_{{p_{i} }} }}^{3} )))} } \right)} \right)} } \right)}}} \right). \\ \end{aligned} $$

Then

$$ \begin{aligned} & \frac{1}{{{}^{n}c_{r} }}\mathop {\,\tilde{ \oplus }\,}\limits_{{1 \le p_{1} < p_{2} < \cdots < p_{r} \le n}} \left( {\tilde{ \otimes }_{i = 1}^{r} w_{{p_{i} }} \alpha_{{p_{i} }} } \right) \\ & \quad = F\left( {\sqrt[3]{{1 - \varphi^{ - 1} \left( {\frac{1}{{{}^{n}c_{r} }}\varphi \left( {1 - \left( {1 - \varphi^{ - 1} \left( {\sum\limits_{{1 \le p_{1} < p_{2} < \cdots < p_{r} \le n}} {\varphi \left( {1 - \varphi^{ - 1} \left( {\sum\limits_{i = 1}^{r} {\varphi (1 - \varphi^{ - 1} (w_{{p_{i} }} \varphi (1 - \mu_{{\alpha_{{p_{i} }} }}^{3} )))} } \right)} \right)} } \right)} \right)} \right)} \right)}}} \right., \\ & \quad \quad \left. {\sqrt[3]{{\varphi^{ - 1} \left( {\frac{1}{{{}^{n}c_{r} }}\varphi \left( {\varphi^{ - 1} \left( {\sum\limits_{{1 \le p_{1} < p_{2} < \cdots < p_{r} \le n}} {\varphi \left( {1 - \varphi^{ - 1} \left( {\sum\limits_{i = 1}^{r} {\varphi (1 - \varphi^{ - 1} (w_{{p_{i} }} \varphi (\nu_{{\alpha_{{p_{i} }} }}^{3} )))} } \right)} \right)} } \right)} \right)} \right)}}} \right) \\ & \quad = F\left( {\sqrt[3]{{1 - \varphi^{ - 1} \left( {\frac{1}{{{}^{n}c_{r} }}\sum\limits_{{1 \le p_{1} < p_{2} < \cdots < p_{r} \le n}} {\varphi \left( {1 - \varphi^{ - 1} \left( {\sum\limits_{i = 1}^{r} {\varphi (1 - \varphi^{ - 1} (w_{{p_{i} }} \varphi (1 - \mu_{{\alpha_{{p_{i} }} }}^{3} )))} } \right)} \right)} } \right)}}} \right., \\ & \quad \quad \left. {\sqrt[3]{{\varphi^{ - 1} \left( {\frac{1}{{{}^{n}c_{r} }}\sum\limits_{{1 \le p_{1} < p_{2} < \cdots < p_{r} \le n}} {\varphi \left( {1 - \varphi^{ - 1} \left( {\sum\limits_{i = 1}^{r} {\varphi (1 - \varphi^{ - 1} (w_{{p_{i} }} \varphi (\nu_{{\alpha_{{p_{i} }} }}^{3} )))} } \right)} \right)} } \right)}}} \right). \\ \end{aligned} $$

Hence

$$ \begin{aligned} & {\text{FFACWMSM}}_{{{\text{AC}}}}^{(r)} (\alpha_{1} ,\alpha_{2} , \ldots ,\alpha_{n} ) \\ & \quad = \left( {\frac{1}{{{}^{n}c_{r} }}\mathop {\,\tilde{ \oplus }\,}\limits_{{1 \le p_{1} < p_{2} < \cdots < p_{r} \le n}} \left( {\tilde{ \otimes }_{i = 1}^{r} w_{{p_{i} }} \alpha_{{p_{i} }} } \right)} \right)^{\frac{1}{r}} \\ & \quad = F\left( {\sqrt[3]{{\varphi^{ - 1} \left( {\frac{1}{r}\varphi \left( {1 - \varphi^{ - 1} \left( {\frac{1}{{{}^{n}c_{r} }}\sum\limits_{{1 \le p_{1} < p_{2} < \cdots < p_{r} \le n}} {\varphi \left( {1 - \varphi^{ - 1} \left( {\sum\limits_{i = 1}^{r} {\varphi (1 - \varphi^{ - 1} (w_{{p_{i} }} \varphi (1 - \mu_{{\alpha_{{p_{i} }} }}^{3} )))} } \right)} \right)} } \right)} \right)} \right)}}} \right., \\ & \quad \quad \left. {\sqrt[3]{{1 - \varphi^{ - 1} \left( {\frac{1}{r}\varphi \left( {1 - \varphi^{ - 1} \left( {\frac{1}{{{}^{n}c_{r} }}\sum\limits_{{1 \le p_{1} < p_{2} < \cdots < p_{r} \le n}} {\varphi \left( {1 - \varphi^{ - 1} \left( {\sum\limits_{i = 1}^{r} {\varphi (1 - \varphi^{ - 1} (w_{{p_{i} }} \varphi (\nu_{{\alpha_{{p_{i} }} }}^{3} )))} } \right)} \right)} } \right)} \right)} \right)}}} \right). \\ \end{aligned} $$

The following results are readily followed from Theorem 4.2:

### **Proposition 4.1**

(Idempotency) *Let* $\alpha_{j} = F(\mu_{{\alpha_{j} }} ,\nu_{{\alpha_{j} }} )\,\,(j \in \Upsilon_{n} )$ *be a collection of FFNs such that* $\alpha_{j} = \alpha_{0} = F(\mu_{{\alpha_{0} }} ,\nu_{{\alpha_{0} }} )\,\,\forall \,j \in \Upsilon_{n}$, *then* ${\text{FFACWMSM}}_{{{\text{AC}}}}^{(r)} (\alpha_{1} ,\alpha_{2} , \ldots ,\alpha_{n} ) = \alpha_{0} .$

### **Proposition 4.2**

(Commutativity) *If* $\alpha^{\prime}_{j}$ *is any permutation of* $\alpha_{j} \,\,(j \in \Upsilon_{n} ),$ *then* ${\text{FFACWMSM}}_{{{\text{AC}}}}^{(r)} (\alpha_{1} ,\alpha_{2} , \ldots ,\alpha_{n} ) = {\text{FFACWMSM}}_{{{\text{AC}}}}^{(r)} (\alpha^{\prime}_{1} ,\alpha^{\prime}_{2} , \ldots ,\alpha^{\prime}_{n} ).$

### **Proposition 4.3**

(Monotonicity) *Let* $\alpha_{j} = F(\mu_{{\alpha_{j} }} ,\nu_{{\alpha_{j} }} )\,\,(j \in \Upsilon_{n} )$ *and* $\alpha^{\prime}_{j} = F(\mu_{{\alpha^{\prime}_{j} }} ,\nu_{{\alpha^{\prime}_{j} }} )\,\,(j \in \Upsilon_{n} )$ *be two sets of FFNs*. *With the aid of the* ${\text{FFACWMSM}}_{{{\text{AC}}}}^{(r)}$ *operator*, *we assume that* ${\text{FFACWMSM}}_{{{\text{AC}}}}^{(r)} (\alpha_{1} ,\alpha_{2} , \ldots ,\alpha_{n} ) = F(\mu_{\alpha } ,\nu_{\alpha } )$ *and* ${\text{FFACWMSM}}_{{{\text{AC}}}}^{(r)} (\alpha^{\prime}_{1} ,\alpha^{\prime}_{2} , \ldots ,\alpha^{\prime}_{n} ) = F\left( {\mu_{{\alpha^{\prime}}} ,\,\nu_{{\alpha^{\prime}}} } \right).$ *If* $\mu_{{\alpha_{j} }} \le \mu_{{\alpha^{\prime}_{j} }} \,{\text{and}}\,\,\nu_{{\alpha_{j} }} \ge \nu_{{\alpha^{\prime}_{j} }}$ *holds for all* $j \in \Upsilon_{n}$, *then we have* $\mu_{\alpha } \le \mu_{{\alpha^{\prime}}} \,\,{\text{and}}\,\,\nu_{\alpha } \ge \,\nu_{{\alpha^{\prime}}}$.

## Proposed Fermatean fuzzy COPRAS (FF-COPRAS) method

**Fig. 1:**
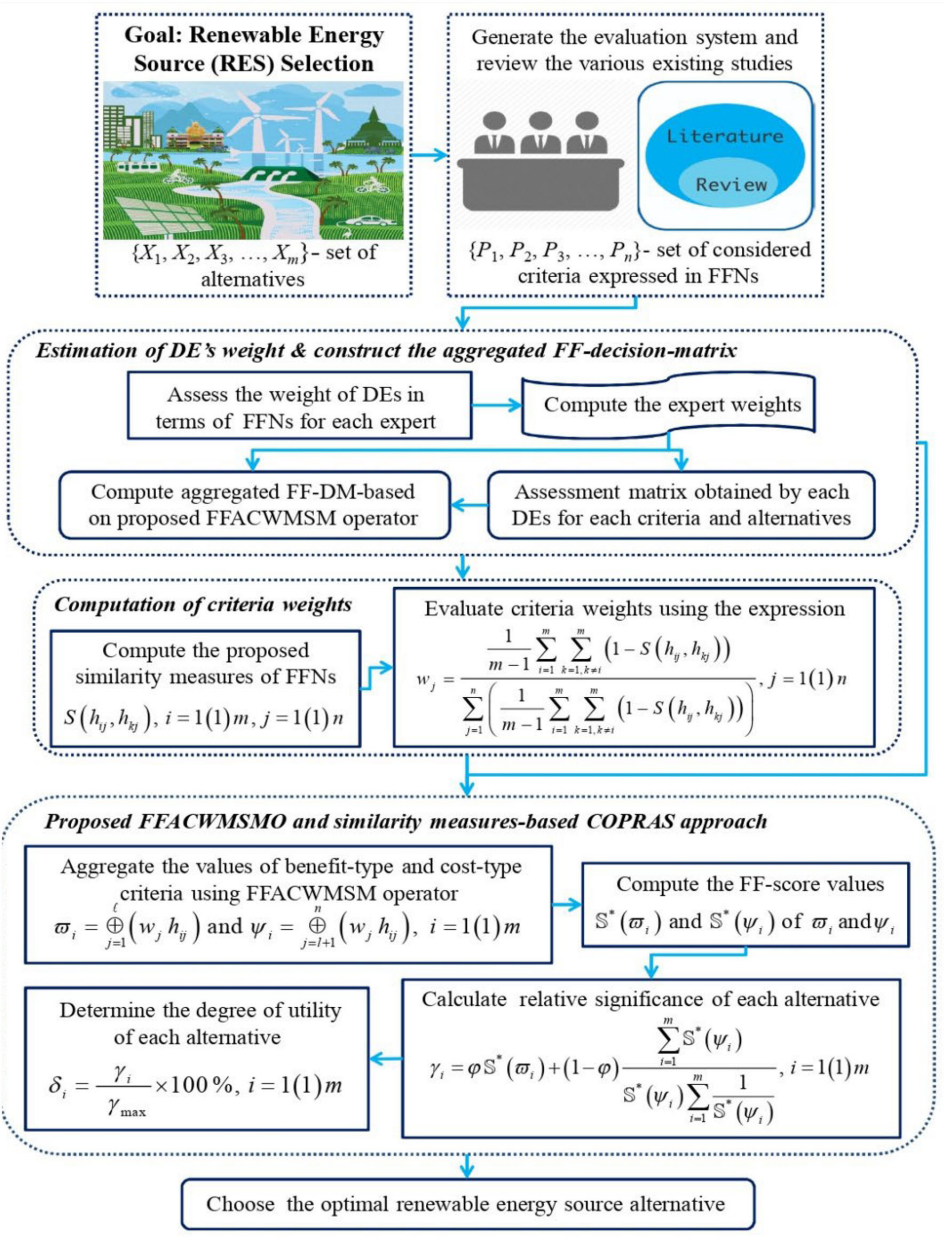
Graphical representation of proposed FF-COPRAS framework

This section presents a methodology for choosing the most appropriate alternative concerning several criteria under the Fermatean fuzzy environment (Fig. 1). The FF-COPRAS method is proposed by utilizing the Fermatean fuzzy Archimedean copula weighted Maclaurin mean operator, score function, and similarity measure from a Fermatean fuzzy perspective. A detailed description of the FF-COPRAS method is shown as

**Step 1:** Define the alternative and criteria.

For an MCDM problem, assume $X = \left\{ {X_{1} ,X_{2} , \ldots ,X_{m} } \right\}$ be a set of options and $P = \left\{ {P_{1} ,P_{2} , \ldots ,P_{n} } \right\}$ be a set of criteria. A panel of DMEs $E = \left\{ {c_{1} ,c_{2} , \ldots ,c_{l} } \right\}$ provides their opinions on each option $X_{i}$ by means of each criterion $P_{j}$ in the form of FFNs. Consider that ${\mathbb{Z}}^{\left( t \right)} = \left( {\varsigma_{ij}^{\left( t \right)} } \right)_{m\, \times \,n}$ be the “*Fermatean fuzzy decision matrix *(*FF-DM*)” provided by the DMEs, in which $\varsigma_{ij}^{(t)}$ refers to the assessment of an option *X*_*i*_ with respect to a criterion $P_{j}$ in the form of FFNs given by $t{\text{th}}$ DME.

**Step 2:** Compute the weights of DMEs.

Since different DMEs may come from different branches with dissimilar backgrounds and areas of interest, therefore, each DME has given a weight $\wp_{t}$ satisfying $\sum\nolimits_{t = 1}^{l} {\wp_{r} } = 1$ and $\wp_{t} \ge 0$ to reflect his/her significance in the assessment of alternatives. To find out the weight values of DMEs, the formula is as follows:

10 $$ \wp_{t} \, = \,\tfrac{{\left( {\mu_{t}^{3} \, + \,\pi_{t}^{3} \, \times \,\left( {\frac{{\mu_{t}^{3} }}{{\mu_{t}^{3} \, + \,\nu_{t}^{3} }}} \right)} \right)}}{{\sum\nolimits_{t\, = \,1}^{\ell } {\left( {\mu_{t}^{3} \, + \,\pi_{t}^{3} \, \times \,\left( {\frac{{\mu_{t}^{3} }}{{\mu_{t}^{3} \, + \,\nu_{t}^{3} }}} \right)} \right)} }}. $$

**Step 3:** Obtain the aggregated FFDM (A-FFDM).

To find the A-FFDM, entire individual decision matrices must be merged into a group along with the DMEs’ judgments. For this purpose, the Fermatean fuzzy Archimedean copula weighted MSM operator is applied and then $M = \left( {\upsilon_{ij} } \right),$ $i = 1,2, \ldots ,m,$ $j = 1,2, \ldots ,n$ is the required A-FFDM, where

11 $$ \upsilon_{ij} = {\text{FFACWMSM}}_{{{\text{AC}}}}^{(r)} (\varsigma_{ij}^{\left( 1 \right)} ,\varsigma_{ij}^{\left( 2 \right)} , \ldots ,\varsigma_{ij}^{\left( t \right)} ) = \left( {\frac{1}{{{}^{t}c_{r} }}\mathop {\,\tilde{ \oplus }\,}\limits_{{1 \le p_{1} < p_{2} < \cdots < p_{r} \le t}} \left( {\tilde{ \otimes }_{y = 1}^{r} \Psi_{{p_{y} }} \varsigma_{ij}^{{\left( {p_{y} } \right)}} } \right)} \right)^{\frac{1}{r}} . $$

**Step 4:** Assess the weights of the criteria.

An innovative formula to compute the importance ratings of criteria has been introduced by means of the proposed similarity measure. First, we assume that each criterion has diverse importance. Let $w = \left( {w_{1} ,w_{2} , \ldots ,w_{n} } \right)^{{\text{T}}}$ such that $\sum\nolimits_{j = 1}^{n} {w_{j} } \, = 1$ and $\,w_{j} \in \left[ {0,\,\,1} \right]$ be the weight vector of the criteria set *P*. To find the weight of the criteria, the following weighting procedure is employed:

12 $$ \begin{aligned} w_{j} &= \frac{{\frac{1}{m - 1}\sum\nolimits_{i = 1}^{m} {\sum\nolimits_{k = 1,\,k \ne i}^{m} {\left( {1 - S\left( {\upsilon_{ij} ,\upsilon_{kj} } \right)} \right)} } }}{{\sum\nolimits_{j = 1}^{n} {\left( {\frac{1}{m - 1}\sum\nolimits_{i = 1}^{m} {\sum\nolimits_{k = 1,\,k \ne i}^{m} {\left( {1 - S\left( {\upsilon_{ij} ,\,\upsilon_{kj} } \right)} \right)} } } \right)} }}, \\ & \quad j = 1\left( 1 \right)n. \end{aligned} $$

**Step 5:** Sum of attribute values based on beneficial and non-beneficial types.

In the present step, each option is articulated with the sum of the maximizing criterion $\varpi_{j}$ and minimizing criterion $\psi_{j} .$ To find the values of $\varpi_{j}$ and $\psi_{j} ,$ the following procedures are implemented based on FFACWMSM operator:

13 $$ \varpi_{i} = {\text{FFACWMSM}}_{{{\text{AC}}}}^{(r)} (\varsigma_{i1} ,\varsigma_{i2} , \ldots ,\varsigma_{i\ell } ) = \left( {\frac{1}{{{}^{\ell }c_{r} }}\mathop {\,\tilde{ \oplus }\,}\limits_{{1 \le p_{1} < p_{2} < \cdots < p_{r} \le \ell }} \left( {\tilde{ \otimes }_{y = 1}^{r} w_{{p_{y} }} \varsigma_{{ip_{y} }} } \right)} \right)^{\frac{1}{r}} $$

and

14 $$ \psi_{i} = {\text{FFACWMSM}}_{{{\text{AC}}}}^{(r)} (\varsigma_{i(\ell + 1)} ,\varsigma_{i(\ell + 2)} , \ldots ,\varsigma_{in} ) = \left( {\frac{1}{{{}^{n - \ell }c_{r} }}\mathop {\,\tilde{ \oplus }\,}\limits_{{1 \le p_{1} < p_{2} < \cdots < p_{r} \le n - \ell }} \left( {\tilde{ \otimes }_{y = 1}^{r} w_{{p_{y} }} \varsigma_{{ip_{y} }} } \right)} \right)^{\frac{1}{r}} . $$

In Eqs. (13) and (14), $\ell$ and *n* are the number of beneficial-type and the total criteria and $w_{j}$ is the weight value of *j*th criterion.

**Step 6:** Computation of the “*relative degree *(*RD*)”.

In the following, the RD $\gamma_{i}$ of *i*th option is computed as

15 $$ \gamma_{i} = \varphi \,{\mathbb{S}}^{*} \left( {\varpi_{i} } \right) + \left( {1 - \varphi } \right)\frac{{\mathop {\min }\nolimits_{i} {\mathbb{S}}^{*} \left( {\psi_{i} } \right)\sum\nolimits_{i = 1}^{m} {{\mathbb{S}}^{*} \left( {\psi_{i} } \right)} }}{{{\mathbb{S}}^{*} \left( {\psi_{i} } \right)\sum\nolimits_{i = 1}^{m} {\frac{{\mathop {\min }\nolimits_{i} {\mathbb{S}}^{*} \left( {\psi_{i} } \right)}}{{{\mathbb{S}}^{*} \left( {\psi_{i} } \right)}}} }}. $$

Here, ${\mathbb{S}}^{*} \left( {\varpi_{i} } \right)$ and ${\mathbb{S}}^{*} \left( {\psi_{i} } \right)$ symbolize the score degrees of $\varpi_{i}$ and $\psi_{i} ,$ respectively.

**Step 7:** Evaluate the priority degree.

In accordance with the RD of each option, the priority degree of options is calculated as below:

16 $$ E^{*} = \mathop {\max }\limits_{i} \gamma_{i} ,\quad i = 1,2, \ldots ,m. $$

**Step 8:** Evaluate the “*utility degree *(*UD*)”.

The UD is computed by comparing each option with the optimal one. The range of the degree of utility lies between 0 to 100%. With the use of the following, the degree of utility of each option can be derived:

17 $$ \delta_{i} = \frac{{\gamma_{i} }}{{\,\,E^{ * } }} \times 100\,\% . $$

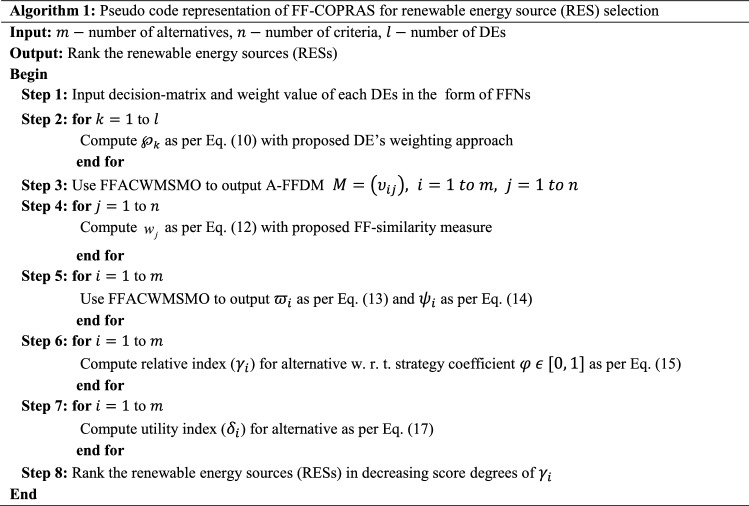


## Case study: renewable energy source (RES) selection

India is one of the world’s fastest emerging economies, with increasing urbanization and an escalating middle class. By 2040, the nation will account for 25% of the growth in global energy usage and will have record growth in energy demand over the next several decades [28]. Gujarat, an emerging renewable energy hub in India, is bordered by “Madhya Pradesh on the east, the Arabian Sea and the Pakistan province on the west, Rajasthan on the north, and Maharashtra and the Union Territories of Diu, Daman, Dadra, and Nagar Haveli on the south”. It is one of the flourishing and resourcefully governed states in India and one of the most popular states in the energy region of India. This state accounts for approximately 9% of total energy demand in India [28]. In 2017–18, Gujarat produced 110,739 MW of renewable power, which is the highest amount as compared to the quantity of different states in India. The “*Gujarat electricity regulatory commission *(*GERC*)” has improved the “*renewable purchase obligation *(*RPO*),” increasing the minimum amount of power to be obtained from “*green energy resources *(*GESs*)” from 10 to 17% over the next 5 years (Policy report, 2019).

**Fig. 2:**
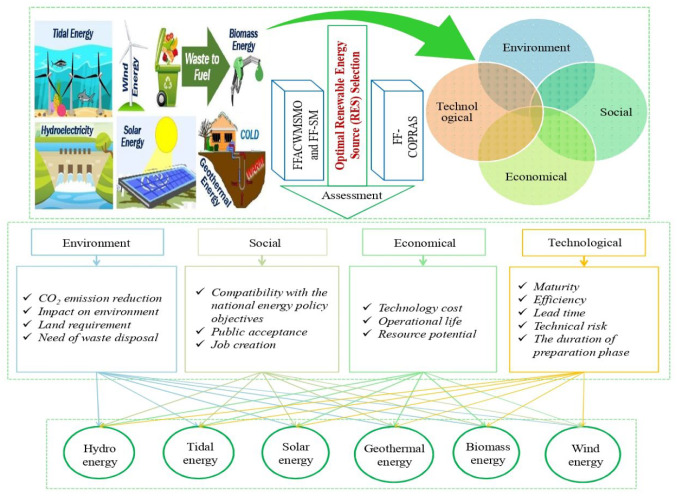
Proposed MCDM model for RES selection

In the current section, we provide a case study of RES selection in Gujarat, India, illustrating the practical use of the developed FF-COPRAS methodology. The systematic evaluation and selection of candidate RES is an imperative topic in Gujarat, India to fulfill the energy needs of customers and further contribute to the country’s economic development. After preliminary analysis, six candidate RESs were selected as the potential alternatives. Furthermore, a panel of three DMEs is created to assign the Fermatean fuzzy values for six RESs over considered attributes. The determination of the index structure, which is complicated with numerous factors considered simultaneously, is a fundamental and critical phase in RES selection problems. To establish an evaluation criteria system, the literature analysis method is adopted in most studies, i.e., the evaluation criteria used in previous literature are fundamental references when DMEs evaluate the considered RESs. Through a literature review, we first summarize 20 representative evaluation criteria. If more than half the literature adopts a certain criterion, then this criterion is identified as an important factor considered in this study. Subsequently, 15 criteria widely used by existing studies were selected and formed a universal evaluation criteria system for RES selection, as shown in Table 1 and Fig. 2. This system is made up of four parts: technical factors, economic factors, environmental factors, and social factors.

**Table 1:** Considered the various aspects and indicators for RES selection

Aspects	Indicators	References
Technological	Maturity (*P*_1_)	Kahraman et al. [39], Shen et al. [74], Alkan and Albayrak [7], Karunathilake et al. [41]
	Efficiency (*P*_2_)	Theodorou et al. [81], Alkan and Albayrak [7], Mishra et al. [55]
	Lead time (*P*_3_)	Kahraman et al. [39]
	Technical risk (*P*_4_)	Kahraman et al. [39]
	The duration of preparation phase (*P*_5_)	Kahraman et al. [39]
Economical	Technology cost(*P*_6_)	Theodorou et al. [81], Shen et al. [74]
	Operational life (*P*_7_)	Burton and Hubacek [15]
	Resource potential (*P*_8_)	Theodorou et al. [81], Shen et al. [74]
Social	Compatibility with the national energy policy objectives (*P*_9_)	Kahraman et al. [39], Deveci et al. [21], Rani et al. [67], Mishra et al. [55]
	Public acceptance (*P*_10_)	Alkan and Albayrak [7], Karunathilake et al. [41], Deveci et al. [21]
	Job creation (*P*_11_)	Kahraman et al. [39], Shen et al. [74], Alkan and Albayrak [7], Rani et al. [67]
Environmental	CO_2_ emission reduction (*P*_12_)	Burton and Hubacek [15], Ahmad and Tahar [2]
	Impact on environment (*P*_13_)	Shen et al. [74], Ahmad and Tahar [2]
	Land requirement (*P*_14_)	Alkan and Albayrak [7], Karunathilake et al. [41], Deveci et al. [21], Rani et al. [67], Mishra et al. [55]
	Need of waste disposal (*P*_15_)	Deveci et al. [21], Rani et al. [67], Mishra et al. [55]

In the data collection stage, the problem is discussed in detail, and the essential and accessible information related to the problem is collected. First, the potential DMEs were selected by the interviews and coordinated in open interviews, and the DMEs had expertise in the field of RESs and decision-making. Therefore, three DMEs were selected, two global and two local to the nation in which the study was conducted. Hence, DMEs collaborated with the authors during the study period.

In the next stage, corresponding to the literature review and open interviews, several sustainability aspects and indicators were collected to select the best RESs. Afterwards, four key aspects were recognized, such as the environmental, technical, economic, and social aspects, and the 15 indicators for selecting the RESs assessment. In addition, open interviews and literature reviews helped us recognize RESs [2, 28, 44, 45, 66]. Meanwhile, six RESs were chosen as the most suitable option in Gujarat, India. In addition, the assessment of RESs is carried out in an FFS setting to deal with uncertain and incomplete data.

**Steps 1**–**2:** Let us consider the weights of DMEs in terms of FFNs, which are given as {(0.80, 0.55, 0.6851), (0.85, 0.50, 0.6390), (0.90, 0.40, 0.5915)}. In accordance with the DMEs’ opinions, the required FFDM is given in Table 2. Since the DMEs’ weights are given in the form of FFNs. To find the crisp DME weights, we have utilized Eq. (10), and therefore, we have {$\wp_{1} =$ 0.3013, $\wp_{2} =$ 0.3317, $\wp_{3} =$ 0.3670}.

**Table 2:** Evaluation ratings of competitive RES selection

	*X*_1_	*X*_2_	*X*_3_	*X*_4_	*X*_5_	*X*_6_
*P*_1_	{(0.60, 0.78), (0.55, 0.72), (0.53, 0.75)}	{(0.62, 0.76), (0.64, 0.75), (0.58, 0.76)}	{(0.57, 0.75), (0.60, 0.70), (0.63, 0.74)}	{(0.62, 0.75), (0.66, 0.71), (0.64, 0.76)}	{(0.55, 0.76), (0.60, 0.74), (0.56, 0.78)}	{(0.65, 0.76), (0.62, 0.72), (0.59, 0.71)}
*P*_2_	{(0.52, 0.77), (0.64, 0.72), (0.56, 0.78)}	{(0.63, 0.76), (0.54, 0.82), (0.67, 0.79)}	{(0.71, 0.66), (0.69, 0.72), (0.67, 0.75)}	{(0.59, 0.78), (0.53, 0.77), (0.62, 0.75)}	{(0.70, 0.65), (0.60, 0.74), (0.58, 0.73)}	{(0.72, 0.60), (0.68, 0.64), (0.69, 0.62)}
*P*_3_	{(0.59, 0.75), (0.56, 0.80), (0.53, 0.77)}	{(0.73, 0.60), (0.71, 0.68), (0.68, 0.78)}	{(0.64, 0.77), (0.65, 0.74), (0.62, 0.76)}	{(0.70, 0.69), (0.62, 0.75), (0.64, 0.73)}	{(0.70, 0.74), (0.64, 0.77), (0.66, 0.72)}	{(0.70, 0.72), (0.64, 0.73), (0.66, 0.70)}
*P*_4_	{(0.60, 0.70), (0.65, 0.75), (0.63, 0.74)}	{(0.67, 0.75), (0.62, 0.78), (0.58, 0.72)}	{(0.62, 0.70), (0.65, 0.78), (0.68, 0.75)}	{(0.64, 0.70), (0.71, 0.75), (0.65, 0.74)}	{(0.64, 0.71), (0.59, 0.75), (0.61, 0.74)}	{(0.64, 0.70), (0.63, 0.75), (0.61, 0.70)}
*P*_5_	{(0.60, 0.73), (0.65, 0.79), (0.64, 0.72)}	{(0.60, 0.78), (0.69, 0.74), (0.65, 0.72)}	{(0.63, 0.76), (0.68, 0.72), (0.67, 0.70)}	{(0.70, 0.65), (0.66, 0.76), (0.68, 0.60)}	{(0.56, 0.78), (0.58, 0.76), (0.63, 0.77)}	{(0.66, 0.78), (0.59, 0.72), (0.61, 0.75)}
*P*_6_	{(0.68, 0.75), (0.63, 0.78), (0.67, 0.79)}	{(0.70, 0.68), (0.65, 0.72), (0.64, 0.75)}	{(0.61, 0.77), (0.62, 0.76), (0.59, 0.81)}	{(0.65, 0.76), (0.63, 0.72), (0.68, 0.75)}	{(0.68, 0.76), (0.55, 0.72), (0.60, 0.74)}	{(0.61, 0.76), (0.65, 0.72), (0.63, 0.74)}
*P*_7_	{(0.68, 0.72), (0.67, 0.78), (0.69, 0.76)}	{(0.76, 0.68), (0.71, 0.73), (0.67, 0.78)}	{(0.74, 0.71), (0.71, 0.64), (0.69, 0.65)}	{(0.67, 0.75), (0.68, 0.77), (0.64, 0.71)}	{(0.64, 0.77), (0.71, 0.64), (0.63, 0.76)}	{(0.66, 0.77), (0.70, 0.62), (0.65, 0.70)}
*P*_8_	{(0.69, 0.73), (0.71, 0.68), (0.67, 0.76)}	{(0.69, 0.74), (0.71, 0.66), (0.73, 0.70)}	{(0.69, 0.71), (0.73, 0.69), (0.72, 0.67)}	{(0.67, 0.74), (0.69, 0.72), (0.63, 0.76)}	{(0.67, 0.78), (0.71, 0.66), (0.73, 0.64)}	{(0.69, 0.74), (0.71, 0.68), (0.72, 0.66)}
*P*_9_	{(0.68, 0.77), (0.71, 0.68), (0.67, 0.76)}	{(0.67, 0.79), (0.63, 0.76), (0.61, 0.74)}	{(0.68, 0.75), (0.64, 0.70), (0.69, 0.74)}	{(0.69, 0.74), (0.63, 0.76), (0.61, 0.72)}	{(0.65, 0.74), (0.63, 0.78), (0.68, 0.79)}	{(0.63, 0.70), (0.61, 0.74), (0.66, 0.74)}
*P*_10_	{(0.71, 0.67), (0.69, 0.73), (0.68, 0.77)}	{(0.63, 0.77), (0.71, 0.69), (0.67, 0.75)}	{(0.73, 0.69), (0.67, 0.74), (0.68, 0.73)}	{(0.71, 0.67), (0.68, 0.73), (0.64, 0.74)}	{(0.72, 0.68), (0.70, 0.63), (0.68, 0.71)}	{(0.72, 0.62), (0.70, 0.61), (0.70, 0.66)}
*P*_11_	{(0.70, 0.63), (0.72, 0.67), (0.67, 0.73)}	{(0.63, 0.71), (0.73, 0.70), (0.68, 0.73)}	{(0.75, 0.63), (0.69, 0.67), (0.77, 0.65)}	{(0.65, 0.78), (0.71, 0.68), (0.62, 0.76)}	{(0.71, 0.66), (0.68, 0.73), (0.71, 0.69)}	{(0.71, 0.68), (0.66, 0.70), (0.71, 0.73)}
*P*_12_	{(0.71, 0.66), (0.70, 0.67), (0.72, 0.63)}	{(0.68, 0.71), (0.67, 0.74), (0.70, 0.69)}	{(0.69, 0.72), (0.70, 0.67), (0.73, 0.68)}	{(0.74, 0.66), (0.71, 0.68), (0.77, 0.65)}	{(0.72, 0.66), (0.73, 0.64), (0.70, 0.68)}	{(0.72, 0.64), (0.73, 0.66), (0.70, 0.67)}
*P*_13_	{(0.69, 0.73), (0.64, 0.77), (0.68, 0.75)}	{(0.60, 0.77), (0.63, 0.75), (0.67, 0.73)}	{(0.76, 0.71), (0.71, 0.69), (0.66, 0.70)}	{(0.69, 0.74), (0.64, 0.73), (0.67, 0.76)}	{(0.71, 0.74), (0.68, 0.71), (0.69, 0.73)}	{(0.71, 0.67), (0.68, 0.72), (0.69, 0.72)}
*P*_14_	{(0.65, 0.74), (0.71, 0.72), (0.69, 0.74)}	{(0.68, 0.73), (0.73, 0.70), (0.70, 0.72)}	{(0.73, 0.69), (0.74, 0.68), (0.70, 0.65)}	{(0.67, 0.71), (0.64, 0.77), (0.69, 0.73)}	{(0.71, 0.69), (0.68, 0.73), (0.67, 0.71)}	{(0.71, 0.66), (0.68, 0.70), (0.67, 0.73)}
*P*_15_	{(0.69, 0.73), (0.66, 0.74), (0.65, 0.76)}	{(0.62, 0.76), (0.64, 0.72), (0.67, 0.71)}	{(0.76, 0.69), (0.71, 0.68), (0.72, 0.70)}	{(0.66, 0.71), (0.62, 0.70), (0.67, 0.74)}	{(0.70, 0.73), (0.68, 0.74), (0.66, 0.71)}	{(0.72, 0.74), (0.67, 0.71), (0.68, 0.70)}

**Step 3:** Combine the distinct opinions of three DMEs in accordance with Eq. (11) and then, A-FFDM is created and shown in Table 3.

**Table 3:** A-FFDM for RES selection

	*X*_1_	*X*_2_	*X*_3_	*X*_4_	*X*_5_	*X*_6_
*P*_1_	(0.559, 0.749, 0.740)	(0.613, 0.757, 0.695)	(0.603, 0.730, 0.732)	(0.641, 0.740, 0.692)	(0.571, 0.761, 0.720)	(0.589, 0.728, 0.742)
*P*_2_	(0.578, 0.757, 0.719)	(0.620, 0.791, 0.644)	(0.689, 0.713, 0.677)	(0.583, 0.766, 0.706)	(0.627, 0.709, 0.735)	(0.696, 0.621, 0.751)
*P*_3_	(0.559, 0.774, 0.712)	(0.706, 0.692, 0.682)	(0.636, 0.756, 0.677)	(0.653, 0.725, 0.699)	(0.666, 0.743, 0.665)	(0.666, 0.716, 0.696)
*P*_4_	(0.628, 0.731, 0.712)	(0.623, 0.749, 0.697)	(0.653, 0.745, 0.675)	(0.668, 0.731, 0.677)	(0.613, 0.734, 0.720)	(0.626, 0.717, 0.729)
*P*_5_	(0.632, 0.746, 0.692)	(0.650, 0.745, 0.678)	(0.662, 0.725, 0.691)	(0.680, 0.667, 0.730)	(0.594, 0.770, 0.694)	(0.620, 0.749, 0.699)
*P*_6_	(0.660, 0.775, 0.627)	(0.662, 0.719, 0.696)	(0.606, 0.781, 0.669)	(0.655, 0.743, 0.676)	(0.612, 0.739, 0.716)	(0.631, 0.739, 0.701)
*P*_7_	(0.680, 0.755, 0.634)	(0.713, 0.733, 0.624)	(0.712, 0.665, 0.701)	(0.663, 0.742, 0.670)	(0.662, 0.723, 0.693)	(0.670, 0.694, 0.714)
*P*_8_	(0.690, 0.724, 0.663)	(0.712, 0.699, 0.668)	(0.715, 0.689, 0.676)	(0.663, 0.741, 0.671)	(0.706, 0.689, 0.685)	(0.708, 0.691, 0.681)
*P*_9_	(0.687, 0.737, 0.652)	(0.636, 0.762, 0.670)	(0.671, 0.730, 0.676)	(0.643, 0.739, 0.691)	(0.655, 0.772, 0.638)	(0.635, 0.728, 0.710)
*P*_10_	(0.693, 0.727, 0.657)	(0.673, 0.736, 0.667)	(0.693, 0.721, 0.664)	(0.676, 0.716, 0.688)	(0.699, 0.674, 0.706)	(0.706, 0.631, 0.735)
*P*_11_	(0.696, 0.680, 0.704)	(0.684, 0.714, 0.681)	(0.740, 0.651, 0.684)	(0.661, 0.740, 0.674)	(0.700, 0.694, 0.685)	(0.694, 0.705, 0.680)
*P*_12_	(0.710, 0.652, 0.714)	(0.684, 0.713, 0.682)	(0.709, 0.689, 0.682)	(0.742, 0.663, 0.669)	(0.716, 0.661, 0.701)	(0.716, 0.658, 0.703)
*P*_13_	(0.670, 0.751, 0.651)	(0.637, 0.749, 0.685)	(0.710, 0.700, 0.670)	(0.667, 0.744, 0.663)	(0.693, 0.726, 0.657)	(0.693, 0.705, 0.682)
*P*_14_	(0.686, 0.733, 0.657)	(0.705, 0.716, 0.656)	(0.723, 0.672, 0.683)	(0.668, 0.737, 0.670)	(0.686, 0.711, 0.683)	(0.686, 0.699, 0.695)
*P*_15_	(0.666, 0.744, 0.664)	(0.646, 0.728, 0.701)	(0.730, 0.690, 0.656)	(0.651, 0.718, 0.708)	(0.679, 0.726, 0.673)	(0.689, 0.715, 0.674)

**Step 4:** Using Eq. (12), the indicator weight with the proposed similarity measure (6) is computed as

*w*_*j*_ = (0.0583, 0.1296, 0.0992, 0.0427, 0.0827, 0.0709, 0.0720, 0.0493, 0.0549, 0.0593, 0.0657, 0.0445, 0.0594, 0.0536, 0.0578)^T^.

**Steps 5**–**8:** Using Eqs. (13)–(27), the values of $\varpi_{i} ,$ ${\mathbb{S}}^{*} \left( {\varpi_{i} } \right),$ $\psi_{i} ,$ ${\mathbb{S}}^{*} \left( {\psi_{i} } \right),$ $\gamma_{i}$ and $\delta_{i}$ of $X_{i} \,\left( {i = \,1,\,2,...,\,6} \right)$ are calculated related to criteria $P_{j} \,\left( {j = 1,2, \ldots ,15} \right)$ and is given in Table 4.

**Table 4:** Computational outcome of FF-COPRAS approach

RES	$\varpi_{i}$	${\mathbb{S}}^{*} \left( {\varpi_{i} } \right)$	$\psi_{i}$	${\mathbb{S}}^{*} \left( {\psi_{i} } \right)$	$\gamma_{i}$	$\delta_{i}$
*X*_1_	(0.566, 0.839, 0.610)	0.295	(0.436, 0.928, 0.490)	0.142	0.2320	100.00
*X*_2_	(0.571, 0.847, 0.591)	0.289	(0.464, 0.918, 0.502)	0.163	0.2181	61.04
*X*_3_	(0.600, 0.818, 0.618)	0.334	(0.457, 0.924, 0.489)	0.154	0.2450	50.90
*X*_4_	(0.561, 0.843, 0.606)	0.288	(0.461, 0.916, 0.512)	0.165	0.2167	54.26
*X*_5_	(0.574, 0.828, 0.624)	0.311	(0.443, 0.924, 0.497)	0.149	0.2360	50.35
*X*_6_	(0.587, 0.807, 0.648)	0.338	(0.452, 0.920, 0.506)	0.157	0.2452	45.72

In accordance with Table 4, the prioritization of the RES options is $X_{6} \, \succ \,X_{3} \, \succ \,X_{5} \, \succ \,X_{1} \, \succ \,X_{2} \, \succ \,X_{4}$ and thus, wing energy (*X*_*6*_) is the best choice.

### Comparative study

In this part of the present study, a comparison is discussed to certify the robustness of the FF-COPRAS methodology. For this, some existing methods, namely, FF-TOPSIS [73] and FF-WPM [72], Karunathilake et al. [41], Rani et al. [67], Deveci et al. [21] and Alkan and Albayrak [7] methods are considered.

#### FF-TOPSIS approach

The FF-TOPSIS has the following computation steps:

**Steps 1**–**4:** Same as the aforementioned method.

**Step 5:** Find out the “*ideal solution *(*IS*)” and “*anti-ideal solution *(*AIS*)” which are computed as

*ξ*^−^ = {(0.571, 0.761, 0.720), (0.620, 0.791, 0.644), (0.706, 0.692, 0.682), (0.668, 0.731, 0.677), (0.680, 0.667, 0.730), (0.662, 0.719, 0.696), (0.663, 0.742, 0.670), (0.663, 0.741, 0.671), (0.636, 0.762, 0.670), (0.673, 0.736, 0.667), (0.661, 0.740, 0.674), (0.684, 0.713, 0.682), (0.637, 0.749, 0.685), (0.668, 0.737, 0.670), (0.646, 0.728, 0.701)} and

*ξ*^+^  = {(0.641, 0.740, 0.692), (0.696, 0.621, 0.751), (0.559, 0.774, 0.712), (0.623, 0.749, 0.697), (0.594, 0.770, 0.694), (0.606, 0.781, 0.669), (0.712, 0.665, 0.701), (0.715, 0.689, 0.676), (0.687, 0.737, 0.652), (0.706, 0.631, 0.735), (0.740, 0.651, 0.684), (0.742, 0.663, 0.669), (0.710, 0.700, 0.670), (0.723, 0.672, 0.683), (0.730, 0.690, 0.656)}, respectively. Next, compute the distances between the alternatives *X*_*i*_ and the AIS and IS over the criterion *P*_*j*_.

**Step 6:** Derive the “*relative closeness index *(*RI*)” to the IS using

18 $$ RI\left( {X_{i} } \right) = \frac{{Y_{i}^{
- } }}{{Y_{i}^{ + } + Y_{i}^{ - } }},
$$

where

$$\begin{aligned} Y_{i}^{ - }& = D\left( {\varsigma_{ij}
,\varsigma^{ - } } \right) = \sum\limits_{j = 1}^{n} {w_{j}}\\
& \!{\sqrt
{\frac{1}{2}\!\left[\! {\left( \!{\left( \!{\mu_{ij} } \right)^{3}\! -\! \left(
{\mu_{j}^{ - } } \!\!\right)^{3} } \!\right)^{2} \!\!+\! \left( {\left(
{\nu_{ij} } \!\right)^{3} \!-\! \left( {f_{j}^{ - } } \!\right)^{3} }\!
\right)^{2} \!+\! \left( \!{\left( {\pi_{ij} } \right)^{3} -\! \left(
{\pi_{j}^{ - } } \!\!\right)^{3} }\!\! \right)^{2} } \right]} } {\text{
and}}\end{aligned} $$

$$ \begin{aligned}Y_{i}^{
+ } =& D\left( {\varsigma_{ij} ,\varsigma^{ + } } \right) =
\sum\limits_{j = 1}^{n} {w_{j}}\\
&{ \sqrt {\frac{1}{2}\!\left[\! {\left(\!
{\left( \!{\mu_{ij} } \right)^{3}\! -\! \left( \!{\mu_{j}^{ + } }\!
\right)^{3} } \!\right)^{2} \!+\! \left(\! {\left(\! {\nu_{ij} }\! \right)^{3}\! -\!
\left( {\nu_{j}^{ + } }\! \right)^{3} } \!\right)^{2}\! +\! \left(\! {\left(\!
{\pi_{ij} } \!\right)^{3} \!- \!\left( \!{\pi_{j}^{ + } } \!\right)^{3} }
\right)^{2} }\! \right]} } .\end{aligned} $$

Then, we obtain RI(*X*_1_) = 0.4853, RI(*X*_2_) = 0.2065, RI(*X*_3_) = 0.6492, RI(*X*_4_) = 0.2587, RI(*X*_5_) = 0.5337 and RI(*X*_6_) = 0.6495.

**Step 7:** Rank the alternatives as $X_{6} \succ X_{3} \succ X_{5} \succ X_{1} \succ X_{4} \succ X_{2} ,$ that is, the most effective option for RES is wind energy (*X*_6_).

#### FF-WPM approach

**Steps 1**–**4:** Same as aforementioned model.

**Step 5:** Since *P*_3_, *P*_4_, *P*_5_, *P*_6_ and *P*_15_ are non-beneficial-type and others are beneficial-type criteria, as a result, we convert A-FFDM into normalized A-FFDM.

**Step 6:** The total significance degree of RESs *X*_*i*_ is defined as $\phi \left( {X_{i} } \right) = \mathop \otimes \nolimits_{j = 1}^{n} \,w_{j} \varsigma_{ij} ,\,\,\,i = 1,2, \ldots ,m.$ Then we find $\phi \left( {X_{i} } \right)$ = {(0.690, 0.699, 0.691), (0.683, 0.720, 0.676), (0.712, 0.684, 0.684), (0.673, 0.714, 0.692), (0.693, 0.689, 0.698), (0.697, 0.670, 0.711)}.

**Step 7:** The score degrees of $\phi \left( {X_{i} } \right)$ for each alternative are computed as ${\mathbb{S}}^{*} \left( {\phi \left( {X_{1} } \right)} \right)$ = 0.494, ${\mathbb{S}}^{*} \left( {\phi \left( {X_{2} } \right)} \right)$ = 0.473, ${\mathbb{S}}^{*} \left( {\phi \left( {X_{3} } \right)} \right)$ = 0.521, ${\mathbb{S}}^{*} \left( {\phi \left( {X_{4} } \right)} \right)$ = 0.470, ${\mathbb{S}}^{*} \left( {\phi \left( {X_{5} } \right)} \right)$ = 0.503 and ${\mathbb{S}}^{*} \left( {\phi \left( {X_{6} } \right)} \right)$ = 0.519. The preference order of options as $X_{3} \succ X_{6} \succ X_{5} \succ X_{1} \succ X_{2} \succ X_{4} .$ Thus, the ranking reflects that the solar energy (*X*_3_) is the best option for given RES selection problem.

**Fig. 3:**
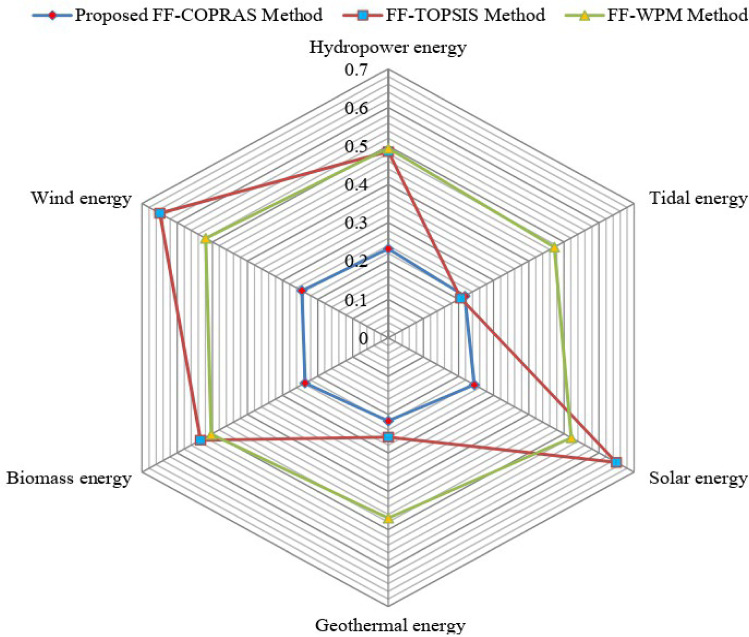
Representation of UD/RI of RES option over different approaches

Based on the FF-TOPSIS approach, the prioritization of the RESs is $X_{6} \succ X_{3} \succ X_{5} \succ X_{1} \succ X_{4} \succ X_{2} ,$ and the most suitable RES option is *X*_6_. Similarly, from the FF-WPM approach, the prioritization of the RES alternative is $X_{3} \succ X_{6} \succ X_{5} \succ X_{1} \succ X_{2} \succ X_{4}$ and hence, the most suitable RES option is *X*_3_. The preference ordering of the alternatives by different methods is graphically depicted in Fig. 3. From the comparative study, we can see that the most suitable RES option, i.e., wind energy (*X*_6_) is equivalent to the developed and FF-TOPSIS methods, whereas the results are slightly different from the FF-WPM method.

The FF-COPRAS approach is found valuable for dealing with quantitative and qualitative decision-making applications with many conflicting indicators. The merits of the developed methodology can be discussed as

- The FFS can handle the uncertainty more precisely than IFS and PFS, so the use of the recently introduced FF-COPRAS method offers a more flexible and reliable way to deal with the uncertain MCDM problems.
- In the developed methodology, the indicator weight is derived with the use of the developed similarity measure-based weighting model, which results in more precise and optimal weights, unlike the arbitrarily chosen indicator weights by DMEs in FF-TOPSIS [73] and FF-WPM [72].
- In the FF-COPRAS methodology, the non-beneficial and beneficial-type indicators are both considered. Consideration of both types of indicators with complex proportions comprises more accurate information in contrast with only treating with non-beneficial or beneficial indicators. It also makes the data easier to read and the results more accurate at the same time.

### Comparison with RES selection methods

To illustrate the validity of the developed approach, we conducted a comparative analysis with three homogeneous MCGDM methods: Karunathilake et al. [41], Rani et al. [67], Deveci et al. [21] and Alkan and Albayrak [7] methods. The advantages of the developed FF-COPRAS methodology are presented in the following:

- The FF-COPRAS approach develops the methodology with the FFSs, unlike Karunathilake et al. [41], Alkan and Albayrak [7], wherein the FSs have been applied, a particular case of the FFSs, and in Rani et al. [67] and Deveci et al. [21], the PFSs and interval-valued IFSs have been applied, also particular cases of the FFSs. Thus, the proposed approach is more suitable for handling uncertainty, indeterminacy, and inconsistent information.
- In this method, the DME’s weight is derived based on a developed procedure, resulting in more realistic significance degrees of DMEs, unlike randomly chosen weights in Karunathilake et al. [41], Alkan and Albayrak [7]. There are two ways to get DME weights: Rani et al. [67] and Deveci et al. [21]. They use a score function-based procedure and an interval-valued intuitionistic fuzzy weighted arithmetic operator.
- Using the proposed similarity measure-based procedure, we can solve the discrepancies that can happen in both objective weight-determining models and subjective weight-determining models, so the results are more accurate and optimum. In Alkan and Albayrak [7], the objective criteria weight-determining model was made by entropy-based procedures on FSs, while in Karunathilake et al. [41], the crisp weights were assumed, leaving no room to handle the uncertainty. In Rani et al. [67], the objective weight of the indicator was obtained by an entropy and divergence measure-based model.

The comparative results are shown in Table 5. We can observe that the best RES alternative wind energy (*X*_6_) in various methods is identical except in the Karunathilake et al. [41] method. Based on the rest of the discussion, the new method has a lot of advantages both in theory and in practice.

**Table 5:** Comparative discussion with extant approaches for different parameters

Parameters	Senapati and Yager [73]	Senapati and Yager [72]	Karunathilake et al. [41]	Rani et al. [67]	Deveci et al. [21]	Alkan and Albayrak [7]	Proposed method
Benchmark	FF-TOPSIS method	FF-WPM method	Fuzzy TOPSIS method	PF-VIKOR method	IVIF–CODAS method	Fuzzy COPRAS and MULTIMOORA	FF-COPRAS
Alternatives/criteria assessments	FFSs	FFSs	FSs	PFSs	IVIFSs	FSs	FFSs
Criteria weight	Assumed	Assumed	Not applicable	Calculated based on information measures	Obtained based on IVIFWAO	Evaluated (entropy-based procedure)	Evaluated (FF-similarity measure-based procedure)
Decision expert weight	Not applicable	Not applicable	Not applicable	Computed	Assumed	Not applicable	Computed
MCDM process type	Single	Single	Single	Group	Group	Group	Group
AOs	Not applicable	FFWGO	Not applicable	FFWAO	Proposed IVIFWAO	Best nonfuzzy performance (BNP) method	Archimedean copula MSM operator
Normalization types	Not applicable	Not applicable	Vector	Vector	Linear	Vector	Linear, vector
Hesitancy degree	Considered	Not considered	Not considered	Considered	Not considered	Not considered	Considered
Optimal RESs	Wind energy (*X*_6_)	Solar energy (*X*_3_)	Hydro energy (*X*_1_)	Wind energy (*X*_6_)	Wind energy (*X*_6_)	Wind energy (*X*_6_)	Wind energy (*X*_6_)

### Sensitivity investigation

**Fig. 4:**
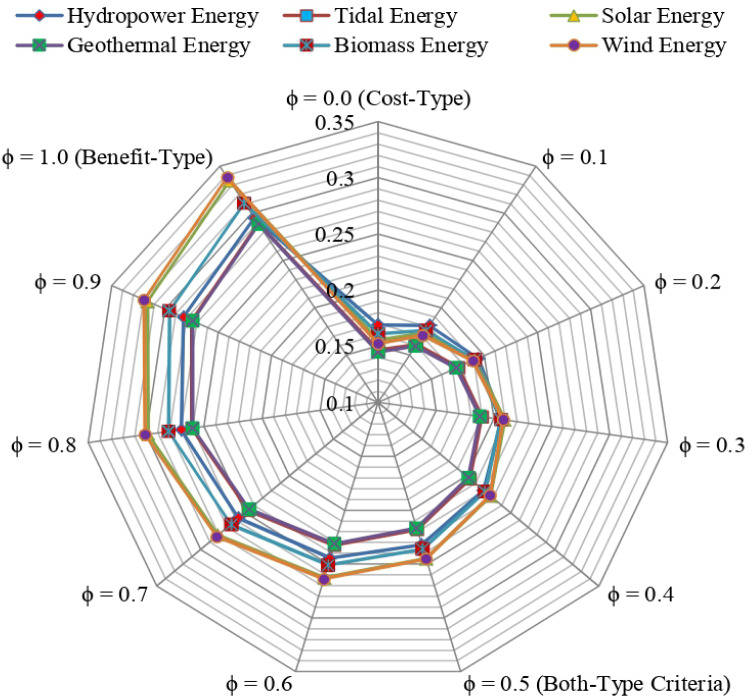
Variation of UD of RES option over diverse parameter (*ϕ*) values

In the section, a sensitivity investigation is discussed to see the effect of diverse values of parameter $\left( \varphi \right)$ on the obtained results. For this purpose, different values of $\varphi \in [0,1]$ are considered for analysis, and the changeable values of $\varphi$ can assist us in evaluating the sensitivity of the FF-COPRAS method. The prioritization of the RES candidates by means of a range of parameter values is shown in Fig. 4. We can see that in Fig. 4, the option *X*_6_ has the highest rank, when $\varphi = 0.5$ to 1.0, while *X*_3_ has the highest rank when $\varphi = 0.0$ to 0.4. While, the RES *X*_4_ has the worst rank when $\varphi = 0.0$ to 1.0. Consequently, it can be observed that the proposed framework has better stability for a range of parameter values. In addition, the criteria weights computed by the developed similarity measure-based formula are preserved to improve the sensitivity of the developed method. Thus, we can understand that the utilization of diverse values of parameter $\varphi$ will enhance the strength of the proposed methodology.

**Fig. 5:**
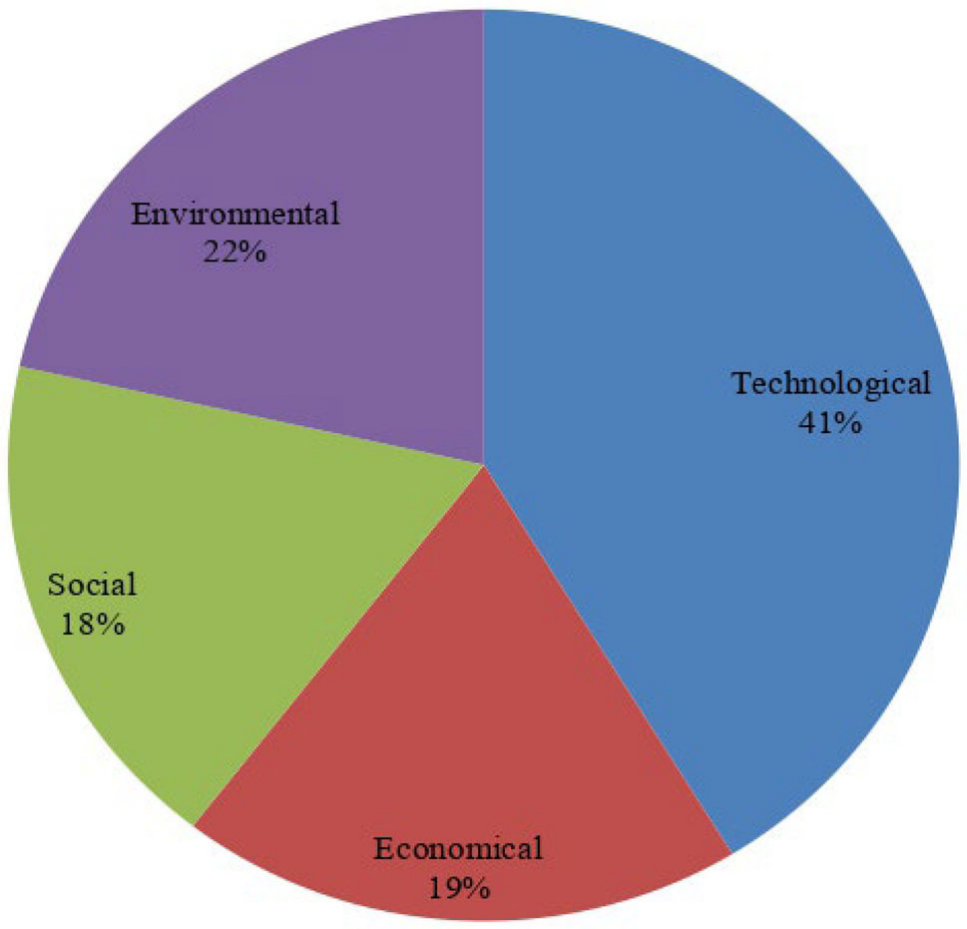
Weight values of various aspects with respect to goal

The outcomes of the work illustrate that technological and environmental indicators are the two most significant aspects, with weight values of 0.41 and 0.22, respectively. The economic aspect is the third most significant aspect, whereas the social aspect is the least significant one. The indicator’s weights are presented in Fig. 5.

**Fig. 6:**
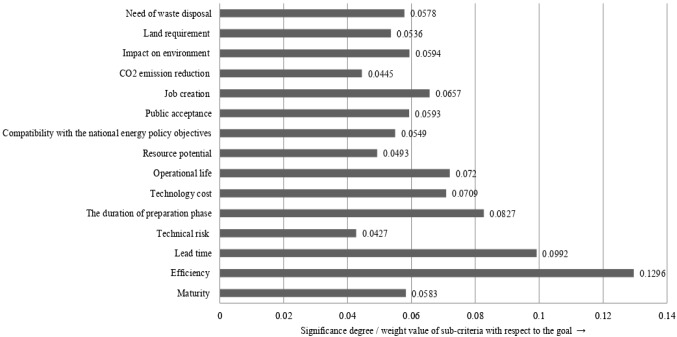
Weight values of diverse indicators with respect to goal

Efficiency has come to be the most important indicator in developing any RES-based generation system. Within the technological criteria, lead time is the second most imperative indicator. From the environmental aspect, the impact on the environment is the most imperative indicator. This priority of impact on the environment over CO_2_ emission reduction, the need for waste disposal and land requirements shows risk-taking performance and the level of acceptance for innovative tools. Operational life has appeared as a more significant indicator in economic terms as compared to technical cost and resource potential. On the social side, job creation is considered crucial as it demonstrates public acceptance and compatibility with the national energy policy objectives indicator. The weight values of indicators with respect to the goal are presented in Fig. 6.

### Implications and discussion

Because traditional MCDM models are ineffective in dealing with uncertain environments and are also incapable of managing the subjectivity of the human mind, a novel FFI-based MCDM method is introduced with the aim of selecting a desirable RES alternative based on several conflicting criteria. In this regard, the first systematic literature survey was conducted to illustrate the key aspects; the outcome was a list of 15 indicators that can be used to assess the candidate RESs. The hierarchical framework is depicted in Fig. 2. Furthermore, to assess the RESs in Gujarat, India with incompatible and conflicting criteria, the FF-COPRAS approach is suggested based on the classical COPRAS method, similarity measure, and Archimedean copula weighted Maclaurin symmetric mean operator within the FFS context.

The weight outcomes show that efficiency (0.1296) was the most significant indicator, followed by the lead time (0.0992), the duration of the preparation phase (0.8270), operational life (0.0720), technology cost (0.0709), job creation (0.0657), impact on the environment (0.0594), public acceptance (0.0593), maturity (0.0583), need for waste disposal (0.0578) and others. Moreover, the comparative analysis with previously developed techniques such as Senapati and Yager [72], Senapati and Yager [73], Karunathilake et al. [41], Rani et al. [67], Deveci et al. [21] and Alkan and Albayrak [7] methods is also specified to elucidate the rationality of the developed FF-COPRAS methodology. The outcome of the comparison showed that wind energy is the most optimum RES candidate among others. Furthermore, the sensitivity investigation is made to see the impact of different values of the parameter. The findings of the sensitivity investigation proved that wind energy is the best one with diverse grading results by means of diverse values of weight $\phi .$ For instance, if $0.0\, \le \,\phi \, \le \,0.2,$ then the prioritization of the RESs determines that hydropower is the most suitable option, followed by biomass, solar, wind, tidal, and geothermal energy. If $0.2\, \le \,\phi \, \le \,1.0,$ then the preferences of the RESs are as follows: wind energy, solar, biomass, hydropower, tidal, geothermal energy.

Major barriers confronted by power generation in producing large-scale RESs and their integration into the extant energy system are as follows: “*Financial Barriers *(*FBs*)”—RESs surfaces *FBs* because of a lack of responsiveness in the technology and the existing resources. “*Political and Policy Barriers *(*PPBs*)”—Strong political views are essential in encouraging the RES projects, both in providing financial assistance as well as policy guidelines. “*Land Availability Challenges *(*LAC*)”—RES plants require large areas of forest land, which is the primary reason for deforestation of forest lands, causing concerns for wildlife or deterioration of the coastal region, threatening tourism. “*Research and Development *(*R&D*)* Cost Barrier *(*R&DCBs*)”—R&D is critical for introducing novel technologies and new production capacity to Gujarat, which requires significant investment. There are the following recommendations to overweigh the barriers:

- The “*Government of India *(*GoI*)” must endorse proper benchmarks and set a few standards for the assessment, stability, and reliability of diverse RESs for greater market saturation.
- RES should be prepared as a prioritization region to raise the availability of funds for the projects and lead to more wide-ranging contributions by commercial banks.
- RES generation will be encouraged in the future by skill development learning conferences for those involved.

With RESs being one of the serious enablers of “*Sustainable Development Goals *(*SDGs*)”, the main attention in the domain is to increase their penetration with the recent power structures. Consequently, the “*United Nations *(*UN*)”, societies, the research community, policymakers, and the private division are working collectively for sustainable RESs for the future [28]. This paper identifies the limitations and barriers to enabling RES integration and discusses policies with recommendations that the government, services, and policymakers can utilize to overpower these barriers and to attain their goal. The government, policymakers, manufacturers, local and international stockholders, and researchers can utilize the results of this work as a valuable recommendation in their scheduling for installing RESs combined projects, not only in Gujarat but also in diverse states of India as well as other regions around the world.

## Conclusions

In the current study, an attempt has been made to introduce a new Fermatean fuzzy decision-making methodology for the assessment and selection of RESs in Gujarat, India. In this respect, the Archimedean copula weighted Maclaurin symmetric mean operator and the similarity measure-based COPRAS method have been proposed in the FFS context. An inclusive review of recent literature has been conducted and then 15 criteria have been selected for evaluating six RESs, including wind, solar, geothermal, biomass, tidal, and hydropower. Among a list of 15 criteria, this study found that efficiency was the most important one. Wind energy was the best option among other RESs.

The current study has treated the three main issues surrounding the FFSs. The axiomatic definition of similarity measure is discussed, and some measures for FFSs are proposed to determine the criteria weighting procedure based on similarity measure. In the second part, the Archimedean copula based Maclaurin symmetric mean operator and its elegant properties are discussed in the FFS context to obtain the aggregate FFI. In the third part, an integrated COPRAS method based on the Archimedean copula based Maclaurin symmetric mean operator and similarity measure is developed, wherein the information about the criteria weight is completely unknown to treat the RESs evaluation problem. According to a comparison study and sensitivity analysis, the results of this study show that the method developed is easy to use, effective, and reliable when used in real-world MCDM problems.

Some limitations of the developed methodology are significant enough to be aware of. The fact that DMEs must be proficient with the preference in order to appropriately use the flexibility and perspective of the FFS setting adds to the implementation complexity. In the following, we present the limitations of the developed MCDM methodology:

- In the FF-COPRAS methodology, all criteria are assumed to be dependent on each other. However, in realistic situations, there are inter-relationships among the criteria.
- An objective weighting procedure is used to find the significant weight value of criteria that are determined from the decision matrices and derived according to the knowledge presented by experts.
- Sustainability should be taken into account more when evaluating RESs as the RES selection problem gets more and more important.

As a future work, we recommend analyzing other information measures, namely, divergence measure, entropy, and correlation coefficient measure, based on MCDM methods with hesitant, q-rung orthopair, and neutrosophic fuzzy extensions. Furthermore, we will continue this work with the hope that the methodology will be found applicable to other issues, such as low carbon supplier selection [52], assessment of the impact of energy and carbon emissions [82], global supply chain management [3], low-carbon tourism strategy evaluation [51], assessment of smart parking for vaccine delivery centers of COVID-19 [38], biomass-to-bioenergy sustainable supply chain network assessment [70] and others.
